# Phytochemistry and pharmacology of sea buckthorn (*Elaeagnus rhamnoides*; syn. *Hippophae rhamnoides*): progress from 2010 to 2021

**DOI:** 10.1007/s11101-022-09832-1

**Published:** 2022-08-11

**Authors:** Jerzy Żuchowski

**Affiliations:** Department of Biochemistry and Crop Quality, Institute of Soil Science and Plant Cultivation, State Research Institute, Czartoryskich 8, 24-100 Puławy, Poland

**Keywords:** Sea buckthorn, *Elaeagnus rhamnoides*, *Hippophae rhamnoides*, Phytochemistry, Pharmacology

## Abstract

Sea buckthorn (*Elaeagnus rhamnoides*; syn. *Hippophae rhamnoides*) is a thorny shrub or a small tree belonging to the Elaeagnaceae family, native to Eurasia. Sea buckthorn fruit is rich in vitamins and minerals, oils from the seeds and fruit flesh find use in medicine and the cosmetic industry or as nutraceutical supplements. Fruit, leaves and other parts of buckthorn have been used in traditional medicine, especially in China, Tibet, Mongolia, and Central Asia countries, and are a rich source of many bioactive substances. Due to its health-promoting and medicinal properties, the plant has been extensively investigated for several decades, and its phytochemical composition and pharmacological properties are well characterized. The years 2010–2021 brought significant progress in phytochemical research on sea buckthorn. Dozens of new compounds, mainly phenolics, were isolated from this plant. Numerous pharmacological studies were also performed, investigating diverse aspects of the biological activity of different extracts and natural products from sea buckthorn. This review focuses on the progress in research on sea buckthorn specialized metabolites made in this period. Pharmacological studies on sea buckthorn are also discussed. In addition, biosynthetic pathways of the main groups of these compounds have been shortly described.

**Graphical abstract:**

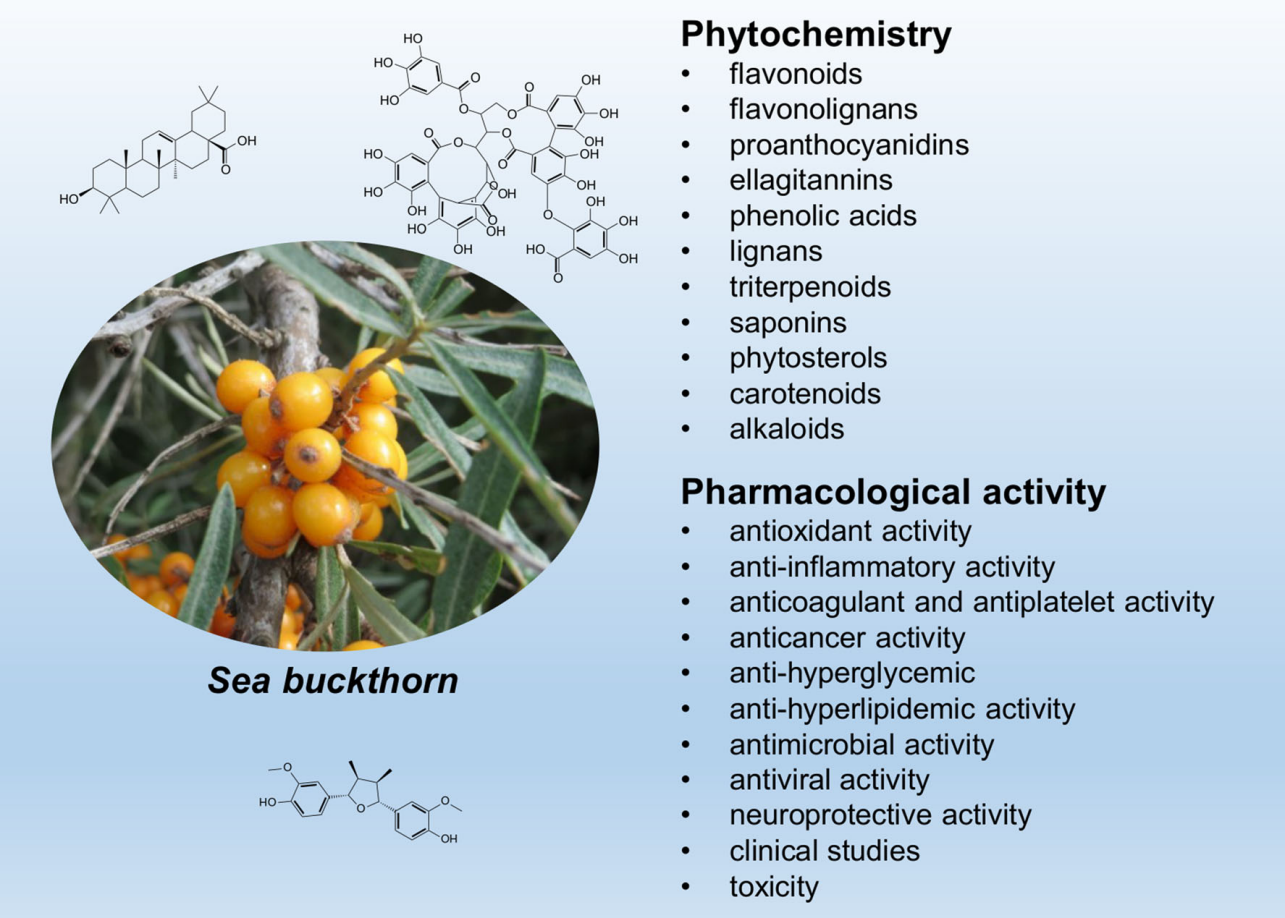

## Introduction

**Fig. 1:**
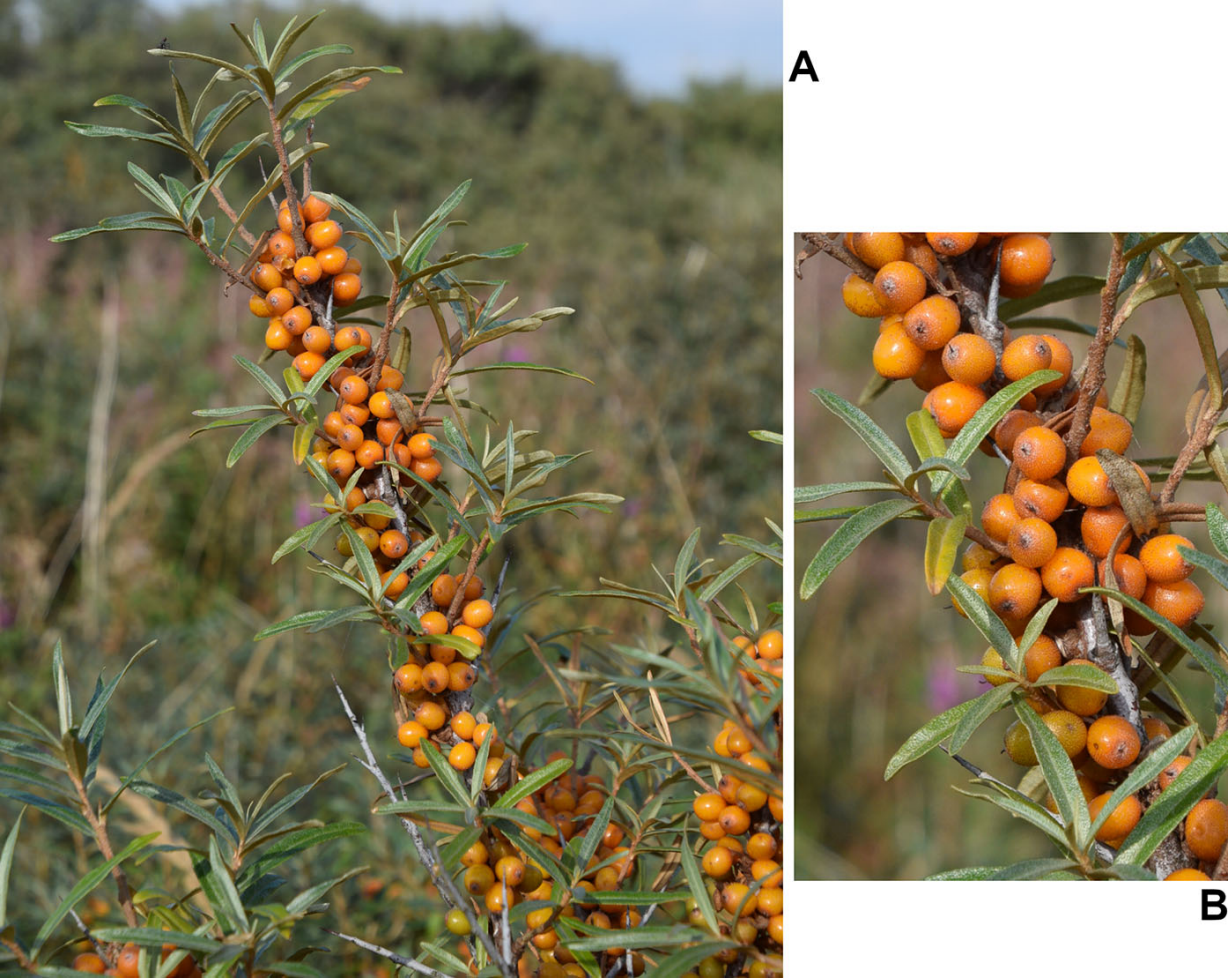
A twig of sea buckthorn (**A**) and its detail (**B**). The photo by Luc.T (Creative Commons Attribution 2.0 Generic license)

Sea buckthorn (*Elaeagnus rhamnoides* (L.) A. Nelson; syn. *Hippophae rhamnoides* L.) is a dioecious, deciduous, thorny shrub or a small tree (Elaeagnaceae), native to Eurasia. It has elongate-oblanceolate or elongate-spathulate leaves, and small, juicy fruit, yellow, orange, or red in colour, with very short (1.5–2 mm) stalks (Fig. 1). The plant lives in a symbiotic association with root-nodule-forming, nitrogen-fixing *Frankia* actinomycetes. It is cold resistant, drought and salt tolerant, with low soil-requirements, which makes it well adapted to growth in different habitats and climatic zones. Sea buckthorn (SB) has become a horticultural crop plant, cultivated in many countries of Europe and Asia, as well as in Canada. Its popularity has been growing over last decades, mainly due to the high nutritional value and medicinal properties of its fruit. It has also found use in the ecological restoration of degraded lands, e.g. for the afforestation of mining heaps. SB fruit is very rich in vitamin C, it contains also significant amounts of vitamin E, vitamin K, β-carotene, as well as mineral elements (mainly P, K, Ca and Mg). The fruit is used in the food industry, to produce juices, jams, syrups etc.. Moreover, both seeds and flesh of the fruit contain valuable oils (100–160 g kg^−1^ seeds, 20–105 g kg^−1^ fresh soft parts), finding use in medicine, cosmetic industry, or as nutraceutical supplements. Fruit, leaves and other parts of SB have been used in traditional medicine, especially in China, Tibet, Mongolia, and Central Asia countries, and the plant is listed in the Chinese Pharmacopeia (Suryakumar and Gupta 2011; Kalia et al. 2011; Letchamo et al. 2018; Ciesarova et al. 2020). Due to its health-promoting and medicinal properties, the plant has been extensively investigated for several decades, and its phytochemical composition and pharmacological properties are well characterized.

The taxonomy of *Hippophae* genus has been a source of controversies. In the first monography (1908) of Elaeagnaceae, only one species was recognized, *H. rhamnoides*, with three subspecies: ssp. *rhamnoides*, ssp. *salicifolia*, and ssp. *tibetana*. Later, Rousi (1971) recognized ssp. *salicifolia*, and ssp. *tibetana* as separate species, and distinguished nine subspecies of *H. rhamnoides*: ssp. *carpatica*, ssp. *caucasica*, ssp. *fluviatilis*, ssp. *gyanthensis*, ssp. *mongolica*, ssp. *rhamnoides*, ssp. *sinensis*, ssp. *turkestanica*, and ssp. *yunnanensis*. This taxonomic division was questioned by some researchers, who raised ssp. *gyanthensis* to the level of species, and distinguished a few more new species. More recent taxonomic studies (www.theplantlist.org) applied the work of A. Nelson (1935), *H. rhamnoides* taxon was synonymized, and its updated name is *Elaeagnus rhamnoides.* Most of the subspecies recognized by Rousi were elevated to species level (i.e. *Hippophae carpatica*, *H. caucasica* etc.), but these taxonomic names are described as unresolved (Letchamo et al. 2018). However, both names, *Hippophae rhamnoides,* and *Elaeagnus rhamnoides,* have been used in recent publications on SB, and the former seems to be far more popular.

The taxonomic issues are far beyond the scope of this narrative review, as well as beyond the knowledge and competences of its author. Readers interested in SB taxonomy may find more details in publications of Letchamo et al. (2018) and Bartish (2016). The aim of this study is to review the progress in the research on SB (regardless of the Latin name, *Hippophae rhamnoides* (including all subspecies), or *Elaeagnus rhamnoides* used in publications) specialized metabolites made from 2010 to 2021. Publications about the biological activity of SB extracts and their constituents have also been described. However, the number of available articles on pharmacological properties of different extracts or natural products from this plant is very large, so that section of the article only highlights some important aspects of research made during the last twelve years. The literature search was performed using Google Scholar, PubMed, and Scopus search engines, with a time limit from 2010 to 2021. Keywords “sea buckthorn” or ‘rhamnoides’ were combined with ‘flavonoids’, ‘ellagitannins’, “phenolic compounds”, ‘phytosterols’, ‘triterpenoids’, ‘alkaloids’, “anti-inflammatory activity”, “anticancer activity”, “antiviral activity”, “clinical trial”, etc. Finally, 97 original articles about SB from this period were included in this review.

## Phytochemistry

The years 2010–2021 brought significant progress in phytochemical research on sea buckthorn; several tenths of new natural products were isolated from fruit, seeds, and leaves of this plant, mainly flavonoids, flavonolignans, and triterpenoid saponins. Apart from the above-mentioned groups, different parts of SB were shown to contain ellagitannins, phenolic acids, lignans, naphthols, naphthoquinones, anthraquinonoids, triterpenoids, phytosterols, carotenoids, volatile compounds, norsesquiterpenoids, and alkaloids. This review is focused on these issues. In the case of ellagitannins and triterpenoids, some older data have been also presented, as these two groups were relatively rarely mentioned in the literature about SB, though they significantly contribute to the bioactivity of this plant. In addition, biosynthetic pathways of the main groups of SB specialized metabolites have been shortly described.

### Flavonoids

**Fig. 2:**
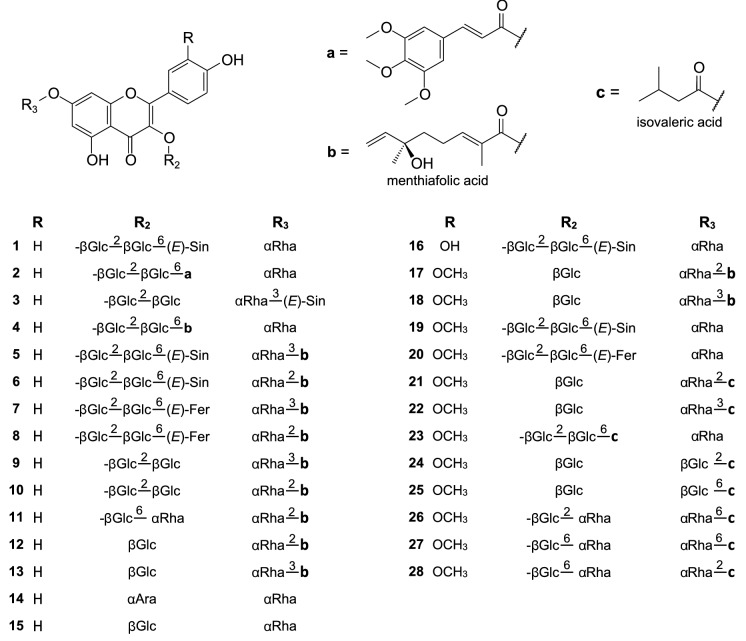
Flavonoids isolated from sea buckthorn

Though sea buckthorn flavonoids were apparently well characterized before 2010, this class of specialized metabolites represents the majority of new compounds isolated from this plant since then. The flavonoid profile of SB fruit comprises mainly diverse simple glycosides of isorhamnetin and quercetin (less frequently kaempferol), though derivatives of myricetin and syringetin were also reported; 3-O-β-glucosides, 3-*O*-rutinosides, 3-*O*-sophorosides, 3-*O*-β-glucoside-7-*O*-α-rhamnosides, 3-*O-*sophoroside-7-*O*-α-rhamnosides, and 3-*O*-rutinoside-7-*O*-α-rhamnosides of the aglycones were the most common. Such compounds were frequently identified in SB fruit extracts by LC–MS, on the basis of their MS spectra, and with the use of standards (e.g., Fang et al. 2013; Pop et al. 2013; Teleszko et al. 2015; Ma et al. 2016; Guo et al. 2017; Tkacz et al. 2021), some of them were also purified from the fruit: 3-*O*-β-sophoroside-7-*O*-α-rhamnosides of kaempferol and isorhamnetin, 7-*O*-rhamnosides of quercetin and isorhamnetin, isorhamnetin 3-*O*-β-galactoside-7-*O*-α-rhamnoside, isorhamnetin 3-*O*-β-glucoside-7-*O*-α-rhamnoside, quercetin 3-*O*-β-glucoside, quercetin 3-*O*-galactoside, rutin, isorhamnetin 3-*O*-β-rutinoside (narcissin), syringetin-3-*O*-glucoside (Fang et al. 2013; Zhang et al. 2018; Żuchowski et al. 2019; Baek et al. 2020). In addition to the above-mentioned compounds, the fruit of SB was shown to contain diverse acylated flavonoids (Fig. 2). Fang et al. (2013) detected, by LC-HRMS, the presence of 13 glycosides of isorhamnetin, quercetin and kaempferol, acylated with coumaric, ferulic, sinapic, and hydroxybenzoic acid, in the extract from died fruit of *H. rhamnoides* Two of them were purified, and identified as new compounds: isorhamnetin 3-*O*-(6-*O*-*E*-sinapoyl-β-D-glucopyranosyl)-(1 → 2)-β-D-glucopyranoside-7-*O*-α-L-rhamnopyranoside (**19**) and isorhamnetin 3-*O*-(6-*O*-*E*-feruloyl-β-D-glucopyranosyl)-(1 → 2)-β-D-glucopyranoside-7-*O*-α-L-rhamnopyranoside (**20**). In addition, several flavonol glycosides acylated with malic acid were tentatively identified. Compound **19**, as well as **1** and **16** (3-*O*-(6-*O*-*E*-sinapoyl-β-D-glucopyranosyl)-(1 → 2)-β-D-glucopyranoside-7-*O*-α-L-rhamnopyranoside of kaempferol and quercetin, respectively) were also found in the fruit of *H. rhamnoides* ssp. *sinensis* (Chen et al. 2013). Another group of novel flavonoids from SB fruit comprise isorhamnetin glycosides acylated with isovaleric acid: isorhamnetin 3-*O*-β-D-glucopyranoside-7-*O*-(2-*O*-isovaleryl)-α-L-rhamnopyranoside (**21**), isorhamnetin 3-*O*-β-D-glucopyranoside-7-*O*-(3-*O*-isovaleryl)-α-L-rhamnopyranoside (**22**), isorhamnetin 3-*O*-[(6-*O*-isovaleryl)-β-D-glucopyranosyl-(1 → 2)]-β-D-glucopyranoside-7-*O*-α-L-rhamnopyranoside (**23**), isorhamnetin 3-*O*-β-D-glucopyranoside-7-*O*-(2-*O*-isovaleryl)-β-D-glucopyranoside (**24**), 3-*O*-β-D-glucopyranoside-7-*O*-(6-*O*-isovaleryl)-β-D-glucopyranoside (**25**), isorhamnetin 3-*O*-α-L-rhamnopyranosyl-(1 → 2)-β-D-glucopyranoside-7-*O*-(6-*O*-isovaleryl)-β-D-glucopyranoside (**26**), isorhamnetin 3-*O*-α-L-rhamnopyranosyl-(1 → 6)-β-D-glucopyranoside-7-O-(6-*O*-isovaleryl)-β-D-glucopyranoside (**27**), isorhamnetin 3-*O*-α-L-rhamnopyranosyl-(1 → 6)-β-D-glucopyranoside-7-*O*-(2-*O*-isovaleryl)-β-D-glucopyranoside (**28**) (Żuchowski et al. 2019). SB seeds, like the fruit, contain many simple flavonol glycosides, but with a (possibly) higher content of compounds with 3 monosaccharide units (Arimboor and Arumughan 2012; Tkacz et al. 2021). Among them, kaempferol 3-*O*-α-L-arabinopyranoside-7-*O*-α-L-rhamnopyranoside (**14**) and 3-*O*-β-D-glucopyranoside-7-*O*-α-L-rhamnopyranoside (**15**) were purified, both reported for the first time from this genus (Zhang et al. 2012). In addition, seeds of *H. rhamnoides* spp. *sinensis* were also shown to contain many acylated flavonoids. This group comprises mainly derivatives of kaempferol 3-*O*-sophoroside-7-*O*-α-L-rhamnoside, and (2*E*)-2,6-dimethyl-6-hydroxy-2,7-octadienoic acid (menthiafolic acid, ( −)-linalool-1-oic acid) (**4**, **9**, **10**), sinapic or 3,4,5-trimethoxycinnamic acid (**1**–**3**), as well as compounds acylated both with menthiafolic acid and sinapic or ferulic acid (5–8) (Zhang et al. 2012; Gao et al. 2013; Chen et al. 2014b; Zhou et al. 2018; Li et al. 2019). Kaempferol 3-*O*-rutinoside-7-*O*-{2-*O*-[(2*E*)-2,6-dimethyl-6-hydroxy-2,7-octadienoyl]}-α-L-rhamnoside (**11**), kaempferol 3-*O*-β-D-glucoside-7-*O*-{2-*O*-[2(*E*)-2,6-dimethyl-6-hydroxy-2,7-octadienoyl]}-α-L-rhamnoside (**12**), and isorhamnetin-3-*O*-β-D-rutinoside-7-*O*-{2-*O*-[(2*E*)-2,6-dimethyl-6-hydroxy-2,7-octadienoyl]}-β-D-glucoside (**18**) were also purified from the seeds (Zhang et al. 2012; Li et al. 2019).

Simple flavonol glycosides of SB leaves are generally similar to those from the fruit and seeds. Several such compounds were purified between 2010–2021, including 3-*O*-β-glucosides, 3-*O*-rutinosides, and 3-*O*-β-glucoside-7-*O*-α-rhamnosides of isorhamnetin, kaempferol and quercetin, as well as 7-*O*-α-rhamnosides of isorhamnetin and quercetin (Kim et al. 2011; Yang et al. 2013; Yuca et al. 2022). Unlike the fruit and seeds, leaves of *E. rhamnoides* were reported to contain tiliroside (kaempferol 3-*O*-(6-*O*-*E*-*p*-coumaroyl)-β-D-glucoside), which was quite frequently isolated (Kim et al. 2011; Yang et al. 2013; Yuca et al. 2022), or detected by LC–MS (Ma et al. 2019; Skalski et al. 2020). Similarly to SB seeds, the leaves contain also flavonol glycosides acylated with menthiafolic acid: **12**, kaempferol 3-*O*-β-D-glucoside-7-*O*-{3-*O*-[2(*E*)-2,6-dimethyl-6-hydroxy-2,7-octadienoyl]}-α-L-rhamnoside (**13**), and isorhamnetin 3-*O*-β-D-glucoside-7-*O*-{2-*O*-[2(*E*)-2,6-dimethyl-6-hydroxy-2,7-octadienoyl]}-α-L-rhamnoside (**17**) (Yang et al. 2013). Similar compounds were also detected by LC-HRMS in a phenolic fraction from the leaves of *E. rhamnoides* (Skalski et al. 2020).

**Fig. 3:**
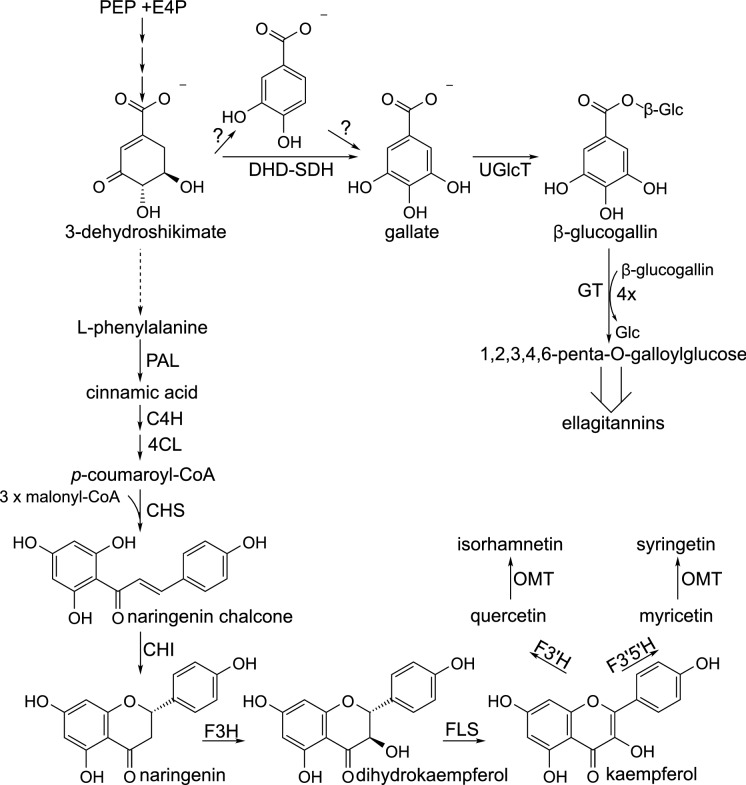
A scheme of biosynthesis of flavonoids and ellagitannins. C4H—cinnamate-4-hydroxylase; 4CL—4-coumarate-CoA ligase; CHS—chalcone synthase; CHI—chalcone isomerase; DHD-SDH—3-dehydroquinate dehydratase/shikimate dehydrogenase; E4P—erythrose 4-phosphate; F3H—flavanone 3-hydroxylase; F3’H—flavonoid 3’-hydroxylase; F3′5H′—flavonoid 3′5′-hydroxylase; FLS—flavonol synthase; OMT—O-methyltransferase; PAL—phenylalanine ammonia lyase; PEP—phosphoenolpyruvate; UGlcT—UDP-glucosyltransferase

Due to the great scientific interest in flavonoids and anthocyanins, as well as proanthocyanidins, their pathway of biosynthesis has been investigated for a long time, and is well characterized (Fig. 3). The first committed step in biosynthesis of SB flavonols is the formation of naringenin chalcone, the result of the condensation of *p*-coumaroyl-CoA (originating from L-phenylalanine) with 3 malonyl-CoA units, catalysed by chalcone synthase. In the next step, the chalcone undergoes stereospeciphic cyclization to (2*S*)-naringenin by chalcone isomerase. Naringenin is subsequently hydroxylated by flavanone 3-hydroxylase to form dihydrokaempferol, and then oxidized to kaempferol by flavonol synthase. Additional hydroxyl groups are added to the B ring of flavonoids by flavonoid 3’-hydroxylase and flavonoid 3′5’-hydroxylase, at the stage of naringenin, dihydrokaempferol, or kaempferol. Biosynthesis of isorhamnetin and syringetin additionally involves methylation, catalysed by S-adenosyl methionine-dependent O-methyl transferases. Glycosylation of the aglycones is catalysed by UDP-glycosyltransferases (Alseekh et al. 2020; Dastmalchi 2021; Liu et al. 2021). Acylation is the final step in biosynthesis of some SB flavonoids, conducted usually by BAHD-acyltransferases (BAHD-ATs), employing CoA-activated acids; however, serine carboxypeptidase-like acyl transferases (SCPL-ATs), using 1-*O*-β-glucose esters as donor molecules, may also be sometimes involved in acylation of flavonoids, as shown for *Brasica napus* (Bontpart et al. 2015; Wilson et al. 2016; Alseekh et al. 2020).

### Flavonolignans

**Fig. 4:**
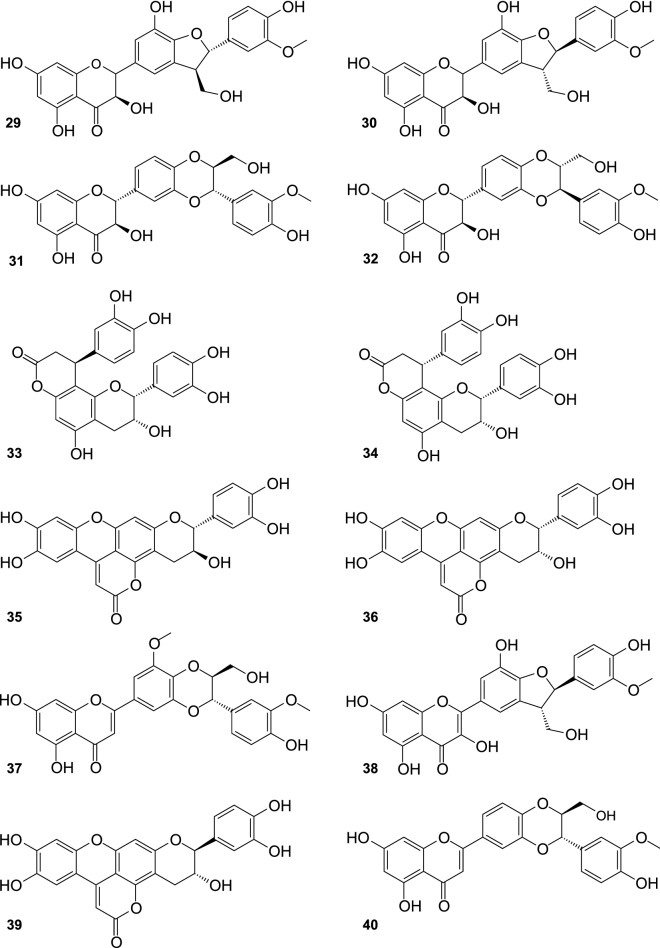

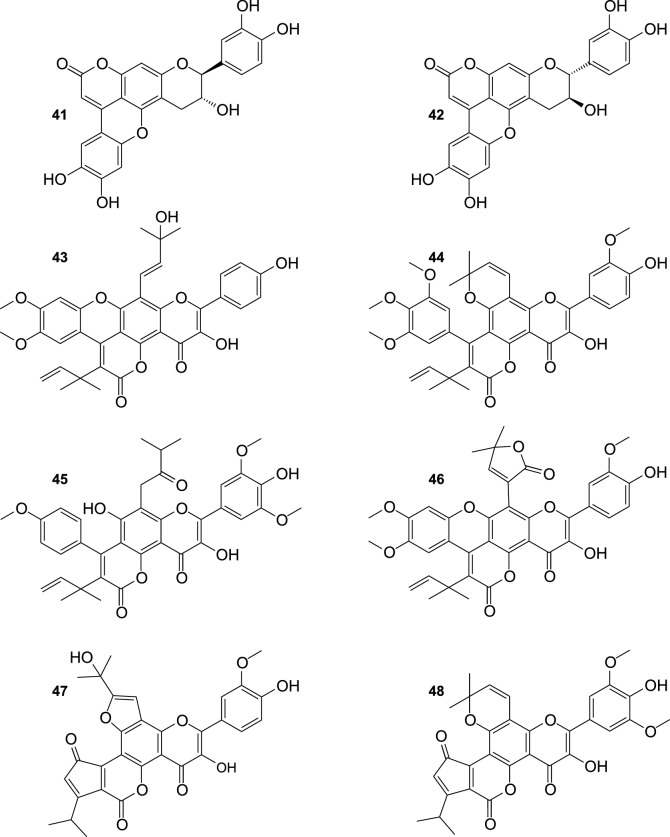

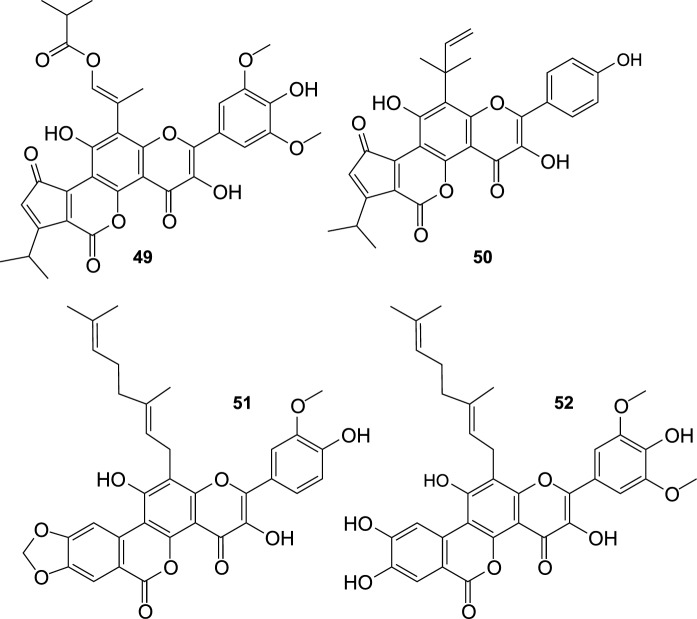
Flavonolignans from the fruit of sea buckthorn continued

Twenty four flavonolignans have been recently purified from the fruit of SB, including 10 new compounds, and 14 substances (Fig. 4) detected for the first time in this plant (Ma et al. 2020). Known compounds included silychristin A (**29**), silychristin B (**30**), silybin B (**31**), silybin A (**32**), cinchonain Ia (**33**) cinchonain Ib (**34**), mururin A (**35**), mururin B (**36**), 5′-methoxyhydnocarpin D (**37**), 2,3-dehydrosilychristin (**38**), ent-mururin A (**39**), hydnocarpin D (**40**), ent-vaccinin A (**41**), and vaccinin A (**42**). New flavonolignans: (*E*)-2-(4-Hydroxyphenyl)-3-hydroxy-8-isopentenol-4″,5″-dimethoxyphenyl-9″-(4‴,5‴-dimethylallyl)dipyranoxanthene-4,10″-dione (**43**), 2-(4′-Hydroxy-3′-methoxyphenyl)-3-hydroxy-12,13-dimethylchromene-3″,4″,5″-trimethoxyphenyl-8″-(4‴,5‴-dimethylallyl)-dipyranoxanthene-4,9″-dione (**44**), 2-(4′-hydroxy-3′,5′-dimethoxyphenyl)-3,7-dihydroxy-8-(12,13-dimethylbutan-10-one)-7″-(4″-methoxyphenyl)-8″-(4‴,5‴-dimethylallyl)dipyranoxanthene-4,9″-dione (**45**), 2-(4′-Hydroxy-3′-methoxyphenyl)-3-hydroxy-8-(13,14-dimethylfuran-12-one)-4″,5″-dimethoxyphenyl-9″-(4‴,5‴-dimethylallyl)-dipyranoxanthene-4,10″-dione (**46**), 2-(4′-Hydroxy-3′-methoxyphenyl)-3-hydroxy-7,8-furan-10-isopropanol-1″,5″-isopropylpentenonepyranochromene-2″,4,6″-trione (**47**), 2-(4′-Hydroxy-3′,5′-dimethoxyphenyl)-3-hydroxy-12,13-dimethylchromene-1″,5″-isopropylpentenonepyranochromene-2″,4,6″-trione (**48**), (*E*)-2-(4′-Hydroxy-3′,5′-dimethoxyphenyl)-3,7-dihydroxy-8-isopropenylated-isobutyrate-1″,5″-isopropylpentenonepyranochromene-2″,4,6″-trione (**49**), 2-(4′-Hydroxyphenyl)-3,7-dihydroxy-8-(12,13-dimethylallyl)-1″,5″-isopropylpentenonepyranochromene-2″,4,6″-trione (**50**), (*E*)-2-(4′-Hydroxy-3′-hydroxyphenyl)-3,7-dihydroxy-8-geranyl-3″,4″-dioxolophenylpyranochromene-4,7″-dione (**51**), and (*E*)-2-(4′-Hydroxy-3′,5′-dihydroxyphenyl)-3,7-dihydroxy-8-geranyl-3″,4″-dihydroxyphenyl-pyrano-chromene-4,7″-dione (**52**).

### Ellagitannins

**Fig. 5:**
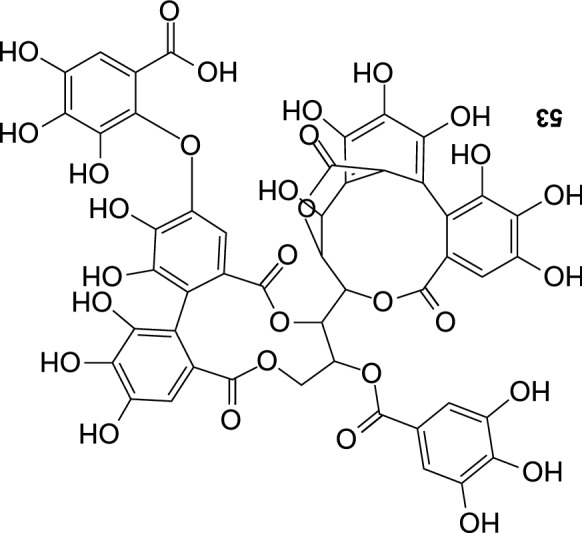
Hippophaenin C

SB leaves are a rich source of ellagitannins, and most of them were identified about 30 years ago (Yoshida et al. 1991). For a long time SB ellagitannins rarely attracted scientific attention, but after 2010 things began to change. These compounds include pedunculagin, casuarinin, casuarictin, strictinin, isostrictinin, tellimagrandin I, hippophaenin A, hippophaenin B, stachyurin, castalagin, vescalagin (Yoshida et al. 1991; Moilanen et al. 2013), elaeagnatin A, pterocarinin A (Moilanen and Salminen 2008), and recently discovered hippophaenin C (**53**; Fig. 5) (Suvanto et al. 2018). UHPLC analyses of Finnish cultivars of SB showed that these compounds might constitute up to ~ 90% of all leaf phenolics, and their total content was estimated as ~ 55–70 mg g^−1^ of the leaf fresh weight (Tian et al. 2017). Stachyurin and casuarinin were identified as dominant ellagitannins in the leaves (Moilanen et al. 2015; Suvanto et al. 2018; Ma et al. 2019).

Gallic acid and glucose are the initial substrates for the biosynthesis of ellagitannins (Fig. 3). Gallic acid derives from the shikimate pathway, and is probably directly synthesized from 3-dehydroshikimic acid, in the reaction catalysed by the dual activity 3-dehydroquinate dehydratase/shikimate dehydrogenase; however, its biosynthesis via protocatechuic acid is not excluded (Salminen 2014; Bontpart et al. 2016; Rock 2017). Formation of 1-*O*-galloyl-β-D-glucose (glucogallin) from gallic acid and UDP-glucose, is the first step of ellagitannin biosynthesis. The molecule is subsequently further galloylated, with the use of other glucogallin molecules as galloyl donors, to form 1, 2, 3, 4, 6-penta-*O*-galloylglucose; these reactions are most probably catalysed by SCPL acyl transferases (Wilson et al. 2016). Further steps of the ellagitannin biosynthesis are only generally known, partly due to a large structural diversity in this group of compounds. The galloyl moieties of the pentagalloylglucose are subsequently subjected to reactions of oxidative coupling (which may be catalysed by laccases), and formation of hexahydroxydiphenoyl units (HHDP), which are a characteristic structural feature of ellagitannins. In this way tellimagrandins II and casuarictin are formed. The addition of more galloyl units, combined with the oxidative coupling reactions (giving HHDP and dehydrohexahydroxydiphenoyl (DHHDP) groups), the removal of gallic acid residues, other modifications, as well as oligomerization lead to formation of plethora of compounds (Ascacio-Valdes et al. 2011; Salminen 2014; Yamashita et al. 2021).

### Other phenolic compounds

**Fig. 6:**
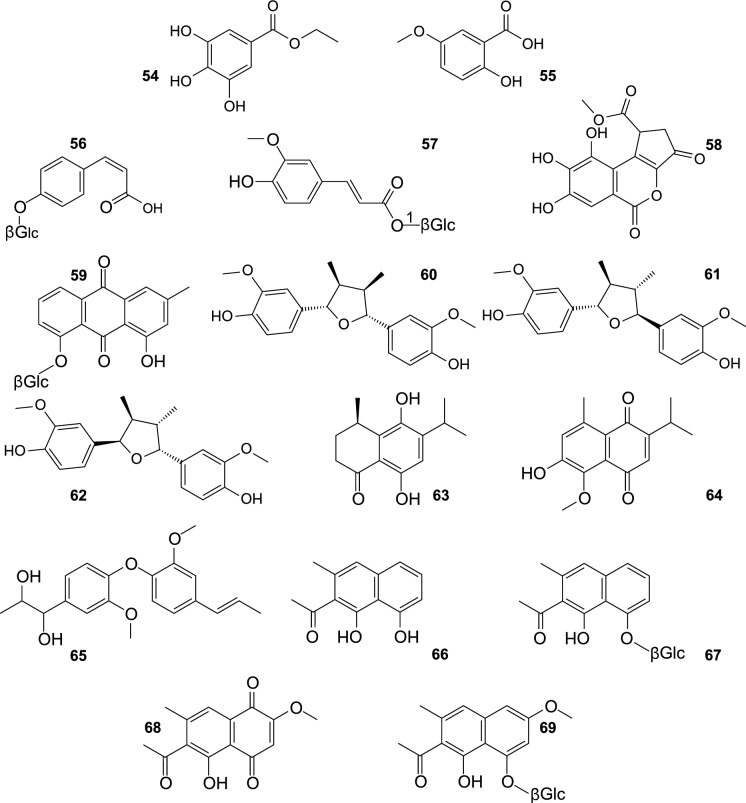
Other phenolic compounds isolated from sea buckthorn.

Like other plants, SB synthetises different phenolic acids, occurring free, or bound as esters and glycosides (Fig. 6). *p*-Hydroxybenzoic, protocatechuic, gallic acid, methyl gallate, ethyl gallate (**54**), and 1-*O*-*E*-feruloyl-β-D-glucose (**57**) were isolated from the leaves of this plant (Kim et al. 2011; Yang et al. 2013; Pandey et al. 2019). The same hydroxybenzoic acids were found in the fruit, as well as *p*-coumaric acid, ferulic acid, 2-hydroxy-5-methoxybenzoic acid (**55**), and *Z*-*p*-coumaric acid 4-*O*-β-glucoside (**56**) (Zhang et al. 2018; Żuchowski et al. 2019; Baek et al. 2020). Moreover, gallic acid derivatives were also purified: methyl brevifolincarboxylate (**58**) from the leaves of SB (Yang et al. 2013), and ellagic acid from the fruit (Zhang et al. 2018).

Catechin, epicatechin, gallocatechin, epigallocatechin, and several B-type proanthocyanidins were previously isolated from SB pomace, seeds and twigs (Rösch et al. 2004; Fan et al. 2007; Yasukawa et al. 2009). Kallio et al. (2014) performed thorough LC–MS analyses of proanthocyanidin fractions, obtained from the fruit of *H. rhamnoides* ssp. *rhamnoides*, and detected more than 60 compounds, with degree of polymerisation from 2 to 11, consisting of (epi)catechin and/or (epi)gallocatechin units.

Apart from flavonolignans, SB was shown to contain also other phenolics rarely associated with this plant, such as chrysophanol 8-*O*-β-D-glucopyranoside, an anthraquinonoid found in seeds of *H. rhamnoides* ssp. *sinensis* (**59**) (Gao et al. 2015), and lignans. Yang et al. (2006) showed that fruit and seeds of *H. rhamnoides* spp. *sinensis*, spp. *rhamnoides* and ssp. *mongholica* contained trace amounts of matairesinol and secoisolariciresinol. Another research group purified nectandrin B (**60**), fragransin A_2_ (**61**), and saucernetindiol (**62**) from a mixture of SB fruit skins, flesh, and seeds (Rédei et al. 2017). Moreover, two new sesquiterpenoids, a phenylpropanoid heterodimer, and four known naphthalenes and naphtoquinones were isolated from SB fruit peels: (R)-6,9-dihydroxy-1-oxo-14-noreudesm-5,7,9-triene (**63**), 2-Hydroxy-1-methoxy-6,9-dioxo-14-noreudesm-1,3,5(10),7-tetraene (**64**), 1-[3-Methoxy-4-(2-methoxy-4-(1E)-propenyl-phenoxy)-phenyl]-propane-1,2-diol (**65**), musizin (**66**), musizin-8-*O*-β-D-glucopyranoside (**67**), 2-methoxystypandrone (**68**), and torachrysone-8-*O*-β-D-glucopyranoside (**69**) (Rédei et al. 2019).

### Triterpenoids and saponins

**Fig. 7:**
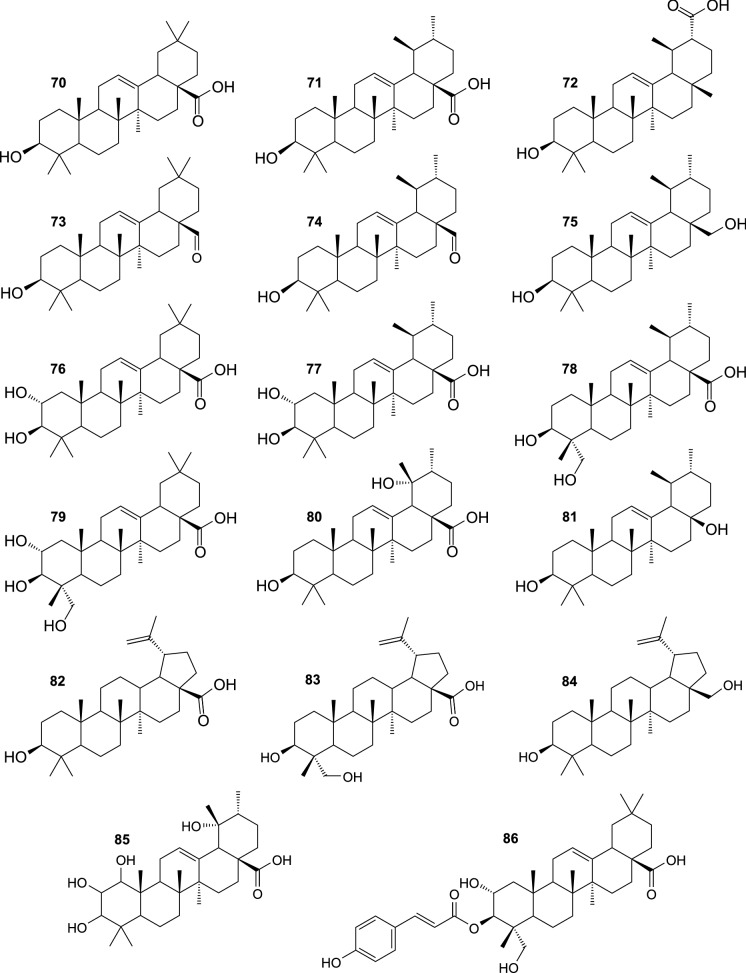
Triterpenoids isolated from sea buckthorn

Phytochemical investigations demonstrated that fruit, branches, and leaves of SB contain pentacyclic triterpenoids, mostly of oleanane- and ursane-type (Fig. 7). These compounds are characterized by diverse biological activities (Parikh et al. 2014; Renda et al. 2021), and contribute to health-promoting and medicinal properties of the plant. Oleanolic acid (**70**) and ursolic acid (**71**) were probably the most frequently isolated from SB triterpenoids (Yang et al. 2007, 2013; Yasukawa et al. 2009; Zheng et al. 2009; Shimoda et al. 2017; Redei et al. 2017). Other triterpenoid compounds, purified from SB, comprise pomolic acid (**80**), dulcioic acid (**72**), corosolic acid (**77**), 23-hydroxyursolic acid (**78**), oleanolic aldehyde (**73**), ursolic aldehyde (**74**), uvaol (**75**), and a nortriterpenoide 28-nor-urs-12-ene-3β,17β-diol (**81**) (Zheng et al. 2009; Yang et al. 2013; Shimoda et al. 2017). Moreover, **70**, **71**, **77**, **80**, maslinic acid (**76**), arjunolic acid (**79**), betulinic acid (**82**), 23-hydroxybetulinic acid (**83**), betulin (**84**), and 1,2,3,19-tetrahydroxy-12-ursen-28-oic acid (**85**) were identified as major SB triterpenoids by LC-HRMS, with the use of authentic standards (Sun et al. 2019; Tkacz et al. 2021). The content of some more hydrophobic compounds, including α-amyrin, β-amyrin, lupeol, erythrodiol, cycloartenol, 24-methylenecycloartanol, and friedelan-3-ol was determined by HPLC, GC, or GC–MS in extracts from SB fruit, or leaves (Teleszko et al., 2015; Madawala et al; 2018; Kukina et al. 2020; Raudone et al. 2021). Some SB triterpenoids occur also in acylated forms. The branch bark of this plant was a source of 3-O-*E*-*p*-coumaroyl oleanolic acid, 3-*O*-*E*-caffeoyl oleanolic acid, 2-*O*-*E*-*p*-coumaroyl maslinic acid, and 2-*O*-*E-*caffeoyl maslinic acid (Yang et al. 2007), while 3-*O*-*E*-*p*-coumaroyl 2,23-dihydroxyoleanolic acid (3-*O*-*E*-*p*-coumaroyl arjunolic acid) (**86**) was isolated from the fruit (Shimoda et al., 2017). In addition, several C_30_H_48_O_4_, C_30_H_48_O_5_, and C_30_H_48_O_6_ triterpenoids, as well as their derivatives acylated with coumaric, ferulic, or caffeic acid, were tentatively identified by LC-HRMS in extracts from SB fruit, twigs, and leaves (Marciniak et al. 2021).

**Fig. 8:**
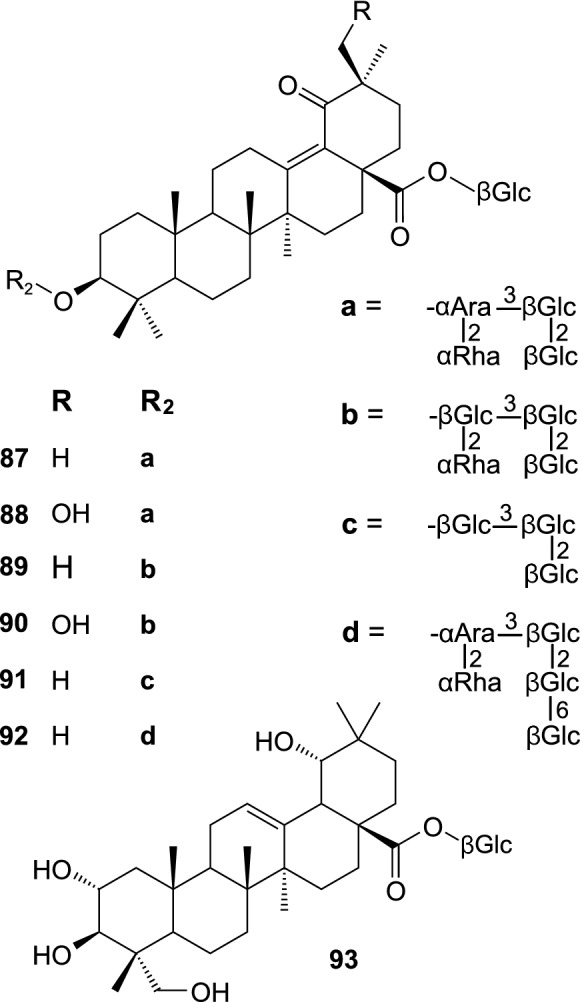
Saponins isolated from sea buckthorn

Saponins constitute a quite recently discovered group of SB constituents (Fig. 8). Six triterpenoid glycosides were isolated from seeds of *H. rhamnoides* ssp. *sinensis*: hippophoside A (3-*O*-[β-D-glucopyranosyl-(1 → 2)-β-D-glucopyranosyl-(1 → 3)]-[α-L-rhamnopyranosyl-(1 → 2)]-α-L-arabinopyranosyl-13-ene-19-one-28-oic acid 28-*O*-β-D-glucopyranosyl ester) (**87**), hippophoside B (3-*O*-[β-D-glucopyranosyl-(1 → 2)-β-D-glucopyranosyl-(1 → 3)]-[α-L-rhamnopyranosyl-(1 → 2)]-α-L-arabinopyranosyl-13-ene-19-one-30-hydroxyolean-28-oic acid 28-*O*-β-D-glucopyranosyl ester) (**88**), hippophoside C (3-*O*-[β-D-glucopyranosyl(1 → 2)-β-D-glucopyranosyl-(1 → 3)]-[α-L-rhamnopyranosyl-(1 → 2)]-β-D-glucopyranosyl-13-ene-19-one-28-oic acid 28-*O*-β-D-glucopyranosyl ester) (**89**), hippophoside D (3-*O*-[β-D-glucopyranosyl-(1 → 2)-β-D-glucopyranosyl-(1 → 3)]-[α-L-rhamnopyranosyl-(1 → 2)]-β-D-glucopyranosyl-13-ene-19-one-30-hydroxyolean-28-oic acid 28-*O*-β-D-glucopyranosyl ester) (**90**) (Chen et al. 2014a), hippophoside E (1-*O*-[(3β)-3-{[β-D-glucopyranosyl-(1 → 2)-β-D-glucopyranosyl-(1 → 3)-β-D-glucopyranosyl]oxy}-19,28-dioxoolean-13(18)-en-28-yl]-β-D-glucopyranose) (**9**1), and hippophoside F (1-*O*-[(3β)-3-{[6-deoxy-α-L-mannopyranosyl-(1 → 2)-[β-D-glucopyranosyl-(1 → 6)-β-D-glucopyranosyl-(1 → 2)-β-D-glucopyranosyl-(1 → 3)]-α-L-arabinopyranosyl]oxy}-19,28-dioxoolean-13(18)-en-28-yl]-β-D-glucopyranose) (**92**) (Gao et al. 2015). Another compound, arjunglucoside I (**93**) has been recently isolated from SB leaves (Yuca et al. 2022). Moreover, 19 triterpenoid glycosides (different from those mentioned above), derivatives of C_30_H_48_O_3_, C_30_H_48_O_4_ and C_30_H_48_O_5_, were tentatively identified by LC-HRMS in a fraction of SB leaf extract (Skalski et al. 2018; Żuchowski et al. 2020); several compounds showing similar *m/z* values (however, reported without any identification and MS2 fragments) were also detected by LC-HRMS in extracts from SB fruit and leaves by Zheng et al. (2019).

**Fig. 9:**
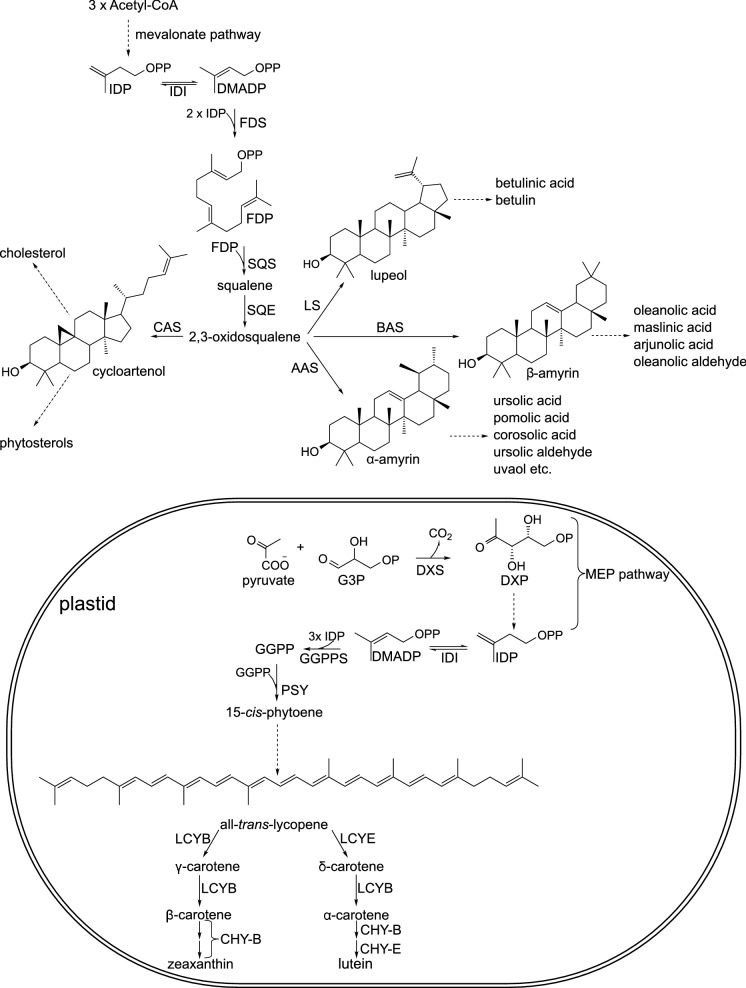
A scheme of biosynthesis of triterpenoids, sterols, and carotenoids. AAS – α-amyrin synthase; BAS – β-amyrin synthase; CAS – cycloartenol synthase; CHY-B – carotene hydroxylase B; CHY-E—carotene hydroxylase E; DMADP—dimethylallyl diphosphate; DXP—1-deoxy-D-xylulose-5-phosphate; DXS—1-deoxy-D-xylulose-5-phosphate synthase; FDP – farnesyl diphosphate; FDS—farnesyl diphosphate synthase; G3P—glyceraldehyde 3-phosphate; GGPP – geranylgeranyl diphosphate; GGPPS—geranylgeranyl diphosphate synthase; IDP – isopentenyl diphosphate; IDI—isopentenyl diphosphate isomerase; LCYB – lycopene cyclase B; LCYE—lycopene cyclase E; LS – lupeol synthase; PSY—phytoene synthase; SQS – squalene synthase; SQE – squalene epoxidase

Precursors of triterpenoids, i.e. isopentenyl diphosphate and dimethylallyl diphosphate, are biosynthesized in cytoplasm, from acetyl-CoA, via mevalonate pathway; two molecules of isopentenyl diphosphate and one molecule of dimethylallyl diphosphate are condensed by farnesyl diphosphate synthase (Fig. 9). Then, two molecules of farnesyl diphosphate (a common substrate for the synthesis of sesquiterpenoids, triterpenoids and sterols) are joined into squalene, by squalene synthase. Squalene is oxidized by squalene monooxygenase (squalene epoxidase) to 2,3-oxidosqualene, the branch point intermediate in biosynthesis of sterols and triterpenoids, which is subsequently cyclised by different oxidosqualene cyclases (e.g. α-amyrin synthase, β-amyrin synthase, lupeol synthase). Synthesis of SB triterpenoid acids involve further oxidation of α-amyrin, β-amyrin, or lupeol by cytochrome P450 enzymes, which catalyse the addition of additional hydroxylic groups, oxidation of OH groups, and transformation of C28 methyl group into the COOH group. The biosynthesis of known SB saponins involves the formation of sugar chains at 3-OH group of the aglycones, and the addition of a single glucose moiety at 28-COOH, catalysed by different UDP-glycosyltransferases (Thimmappa et al. 2014; Rahimi et al. 2019; da Silva Magedans et al. 2021).

### Carotenoids and sterols

SB fruit owes its colour to the presence of carotenoids, and the composition and/or content of these compounds have been often determined. In the described timeframe, many LC–MS analyses of carotenes and xanthophylls in the fruit, oils, and (rarely) leaves of *E. rhamnoides* were reported (e.g. (Giuffrida et al. 2012; Pop et al. 2014; Czaplicki et al. 2017; Ursache et al. 2017; Madawala et al. 2018; Tudor et al. 2019; Tkacz et al. 2020; Seglina et al. 2021). Analyses of extracts of native carotenoids from the fruit of different cultivars of *H. rhamnoides* spp *carpatica* showed the presence of several free carotenoids: α, β, γ, and δ-carotene, *cis* β-carotenes, lycopene and *cis*-lycopene, zeaxanthin, lutein and β-cryptoxantin; however, zeaxanthin, lutein, and β-cryptoxantin occurred also as mono- and diesters of fatty acids (mainly myristic and palmitic acid), and the xanthophyll esters constituted the majority of all carotenoids in the fruit (Giuffrida et al. 2012; Pop et al. 2014). Similar results (though without β-cryptoxantin, and *ci*s-lycopene) were obtained for the fruit oil from the SB cultivar Mara (Tudor et al. 2019). The leaf extracts contained only β-carotene, *cis*-β-carotenes, lutein, zeaxanthin, violaxanthin, and neoxanthin (Pop et al. 2014). Carotenoids are synthesized in plastids (Fig. 9). Their biosynthetic precursors, isopentenyl diphosphate (IPP) and dimethylallyl diphosphate (DMAPP), are produced via 2C-methyl-D-erythritol-4-phosphate (MEP) pathway. Pyruvate and glyceraldehyde-3- phosphate are condensed by 1-deoxy-D-xylulose-5-phosphate synthase (DXS) into 1-deoxy-D-xylulose-5-phosphate, which is subsequently transformed into MEP by 1-deoxy-D-xylulose-5-phosphate reductoisomerase (DXR). Then, MEP is converted, in three enzymatic steps, into 2C-methyl-D-erythritol-2, 4-cyclodiphosphate, oxidized to 1-hydroxy-2-methyl-2-(E)-butenyl-4-diphosphate (HMBPP) by HMBPP synthase. Finally, dehydratation of HMBPP, catalysed by HMBPP reductase leads to formation of IPP and DMAPP. One molecule of DMAPP and 3 molecules of IPP are condensed into geranylgeranyl diphosphate (GGPP) by GGPP synthase. Condensation of two molecules of GGPP into 15-*cis*-phytoene by phytoene synthase (PSY) is the first committed step of carotenoid biosynthesis. The compound is subsequently transformed into all-*trans*-lycopene, in a series of reactions catalysed by phytoene desaturase (PDS), 15-cis-ζ-carotene- isomerase (Z-ISO), ζ-carotene desaturase (ZDS), and carotene isomerase (CRTISO). The synthesis of α-, β-, γ-, and δ-carotenes is associated with formation of β- or ε-ionone rings, catalysed by lycopene cyclases B and E, respectively (LCYB & LCYE). Xanthophylls, such as zeaxanthin, β-cryptoxantin, and lutein, are synthesised from β- and α-carotenes by hydroxylation of one or two ionone rings by carotene β-hydroxylase (CHY-B) and carotene ε-hydroxylase (CHY-E). Carotene hydroxylases B and E are cytochrome P450 enzymes, CYP97A and CYP97C; alternatively, hydroxylation of β-ionone rings may be catalysed by ferrodoxin-dependent nonheme diiron enzymes. A flavin-dependent monooxygenase, zeaxanthin epoxidase (ZEP), participates in biosynthesis of epoxidated xanthophylls, such as violaxanthin (Moise et al. 2014; Llorente 2016).

Sterols and stanols constitute another group of lipophilic compounds, frequently determined in SB fruit and oils. β-Sitosterol was identified as dominant compound, stigmasterol, campesterol, Δ^5^-avenasterol (isofucosterol), Δ^7^-avenasterol, gramisterol, cholesterol, clerosterol, 24-methylencholesterol, citrostadienol, sitostanol, and stigmastanol were also found (Teleszko et al. 2015; Madawala et al. 2018; Shi et al. 2019; Seglina et al. 2021).

As mentioned above, oxidation of squalene to 2, 3-oxidosqualene is a branching point in biosynthesis of triterpenoids and phytosterols (Fig. 9). 2, 3-oxidosqualene is converted to cycloartenol by cycloartenol synthase. Cycloartenol can be reduced by sterol side chain reductase 2 (SSR2) to cycloartanol, and further transformed, in 8 enzymatic steps, into cholesterol. Alternatively, C-24 sterol methyltransferase 1 (SMT1) catalyses transformation of cycloartenol into 24-methylenecycloartenol, which is a starting point for the multistep biosynthesis of diverse phytosterols. For example, the biosynthesis of stigmasterol from 24-methylenexycloartenol occurs via 11 enzymatic reactions, with Δ^7^-avenasterol, Δ^5^-avenasterol, and β-sitosterol as late intermediates (Sonawane et al. 2016; De Vriese et al. 2021).

### Essential oils

SB fruit contains a complex mixture of volatile compounds. Socaci et al. (2013) analysed volatiles from the fruit of 12 different cultivars and wild plants of *H. rhamnoides* ssp. *carpatica* by GC–MS; ethyl 2-methylbutanoate, ethyl 3-methylbutanoate, ethyl hexanoate, 3-methylbutyl 3-methylbutanoate, 3-methylbutyl 2-methylbutanoate, ethyl octanoate, ethyl butanoate, and ethyl benzoate accounted for over 80% of the samples. The samples contained also alcohols (3-methyl-butanol, 1-hexanol), aldehydes (heptanal, benzaldehyde, octanal), ketones (6-methyl-5-hepten-2-one, acetophenone) and terpenes (limonene, *cis*-ocimene). GC–MS analyses of an essential oil from the fruit *H. rhamnoides* ssp. *mongholica* showed the presence of carboxylic acids and their esters (71.45%, mainly 3-methylbutyl benzoate, ethyl hexanoate, ethyl (9*Z*)-hexadecenoate, and 3-methylbutyl 3-methylbutanoate, ethyl octanoate), as well as oxygenated terpenoids (5.12%, mainly isolongifolen-9-one), sesquiterpenes (4.07%, mainly germacrene B, β-maaliene), phenylpropanoids (1.50%, *E*-isoelemicin, β-asarone), alkenes (3.42%) and alkanes (0.29%) (Slynko et al. 2019). These results are in a way similar to those from some older studies (Hirvi et al. 1984; Tiitinen et al. 2006a). In contrast, GC–MS analyses of volatiles from 13 cultivars of SB detected the presence of 26 alcohols (mainly ethanol, propan-1-ol, propan-2-ol, butan-2-ol, pentan-1-ol, pentan-2-ol, 2-methylpropan-1-ol, 2-methylbutan-1-ol, 3-methyl-butan-1-ol, methanol, benzyl alcohol, phenylethanol), 12 aldehydes (mainly acetaldehyde, heptanal, octanal, (*E*)-oct-2-enal), 11 ketones (mainly 3-hydroxybutan-2-one and tridecan-2-one), 9 acids (mainly acetic, propanoic, 2-methylpropanoic,3-methylbutanoic acid), and 11 esters (mainly ethyl octanoate, ethyl butanoate, butyl acetate) (Vitova et al. 2015). These large differences in the reported composition of the analysed samples may be attributed to differences among SB subspecies and cultivars, as well as their growth conditions.

### Other compounds

Commonly occurring constituents of fruits, such as sugars and their derivatives (glucose, fructose, sucrose, ethyl β-glucopyranoside), carboxylic acids (malic, quinic, citric, ascorbic acid) were frequently determined in SB (e.g. Tiitinen et al. 2006a, b; Yang et al. 2011; Zheng et al. 2012). Since 2010, SB fruit cyclitols has been also well characterized, this group includes L-quebrachitol (a major compound), methyl-*myo*-inositol, and *myo*-inositol (Yang et al. 2011; Zheng et al. 2012).

**Fig. 10:**
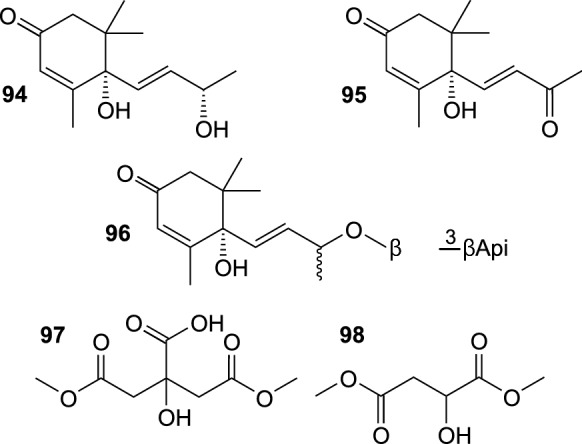
Other aliphatic compounds isolated from sea buckthorn

**Fig. 11:**
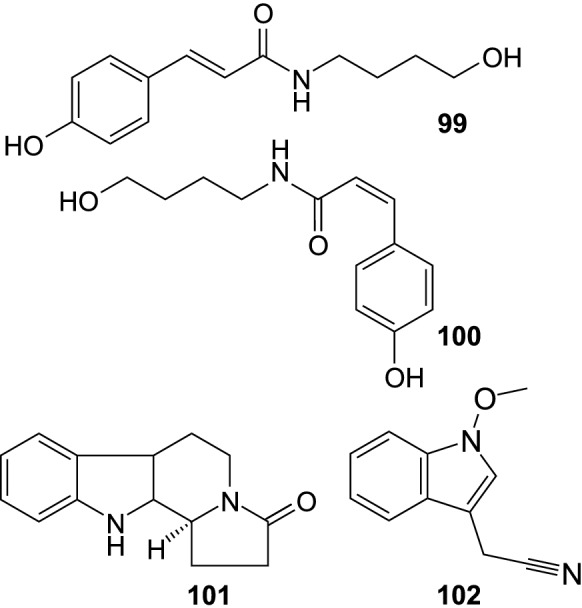
Nitrogen-containing compounds isolated from sea buckthorn

In addition, several other compounds (Fig. 10) were isolated from different parts of SB, including 1,5-dimethyl citrate (**97**), (S)-dimethyl malate (**98**), as well as norsesquiterpenoids vomifoliol (**94**) and ( +)-dehydrovomifoliol (**95**) from SB fruit (Baek et al. 2020; Lee et al. 2021); vomifoliol was also purified from SB leaves (Yang et al. 2013), and vomifoliol 9-*O*-β-D-apiofuranosyl-(1 → 6)-β-D-glucopyranoside (**96**) from the seeds of *H. rhamnoides* ssp. *sinensis* (Gao et al. 2015). Nitrogen-containing specialized metabolites were rarely reported from SB (Fig. 11). In recent years, 4-[(*E*)-*p*-coumaroylamino]butan-1-ol (**99**), 4-[(*Z*)-*p*-coumaroylamino]butan-1-ol (**100**), and a pyridoindole alkaloid hippophamide ((11bS)-1,2,5,6,11,11b-hexahydro-8-hydroxy-3*H*-indolizino[8,7-*b*]indol-3-one) (**101**) were isolated from seeds of *H. rhamnoides* ssp. *sinensis* (OuYang et al. 2015), while caulilexin C (**102**) was found in peels of SB fruit (Rédei et al. 2019). Moreover, a butanol extract from SB twigs was shown to contain significant amounts of tricoumaroyl spermidine, feruloyl dicoumaroyl spermidine, diferuloyl coumaroyl spermidine, and triferuloyl spermidine, which were tentatively identified by LC-HRMS (Skalski et al. 2020).

## Pharmacology

Sea buckthorn is a medicinal plant, used in traditional and conventional medicine, and cultivated for its fruit, rich in health-promoting compounds. For these reasons, the plant has attracted scientific attention for many years, and its pharmacological properties have been studied in many ways. As in the case of many other plants, it seems that antioxidant, anti-inflammatory, anticancer, as well as antimicrobial activity were the most often investigated bioactivities of sea buckthorn. Antidiabetic, anti-hyperlipidemic, hepatoprotective, and neuroprotective properties of the plant were also frequently described. In contrast, new studies on antiviral activity of SB seem to be rare. As it has been mentioned above, the number of publications on the pharmacological activities of SB is very large, and most of them describe properties of SB oils, extracts, or fractions. This section of the article only highlights some important aspects of research published between 2010 and 2021, and is focused (when possible) on activities of compounds isolated from SB. Reports on clinical trials and toxicological studies are also described.

### Antioxidant activity

Antioxidant properties of preparations from different parts of SB are well known, and many aspects of their activity were very frequently investigated, using diverse methods. The amount of available data is enormous, so this compilation will be focused solely on results concerning compounds isolated from this plant, or detected in it. Kim et al. (2011) determined the DPPH^·^ radical scavenging activity of six phenolic compound from SB leaves, tiliroside, 1-feruloyl-β-D-glucopyranoside (57), isorhamnetin 3-*O*-glucoside, quercetin 3-*O*-glucoside, quercetin 3-*O*-glucoside-7-*O*-rhamnoside, and isorhamnetin 3-*O*-rutinoside; the respective EC_50_ values were 5.32, 13.79, 159.20, 1.86, 59.83, and 87.19 µg mL^−1^. The reducing power of the compounds was also investigated; the spectrophotometric measurements for 200 µg mL^−1^ solutions of the compounds gave OD_700_ values: 0.90, 0.71, 0.22, 1.78, 0.20, and 0.24, respectively, while OD_700_ for 200 µg mL^−1^ ascorbic acid was 2.03. DPPH^·^ and ABTS^·^ radical scavenging activity was also measured for another set of SB leaf phenolics. IC_50_ values for DPPH^·^ scavengers were 522 µM for isorhamnetin 3-*O*-glucoside-7-*O*-rhamnoside, 161 µM for isorhamnetin-7-*O*-rhamnoside, 91 µM for quercetin 3-*O*-glucoside, 395 µM for isorhamnetin 3-*O*-rutinoside, 777 µM for isorhamnetin 3-*O*-glucoside, and 14 µM for casuarinin. In the case of ABTS^·^ scavenging activity, the respective IC_50_ were 18, 27, 11, 14, 16, and 2 µM, as well as 558 µM for arjunglucoside I (**93**) (Yuca et al. 2022). Arimboor and Arumughan (2012) investigated several aspects of antioxidant properties of SB leaf extracts, quercetin, kaempferol, isorhamnetin, rutin, and gallic acid. IC_50_ values for DPPH^·^ scavenging activity ranged from 1.6 (gallic acid) to 10.0 µg mL^−1^ (rutin); ABTS^·^ scavenging activity, expressed as TEAC, ranged from 7.5 (rutin) to 36.3 nM (quercetin); IC_50_ values for the hydroxyl radical scavenging were 18.0 (quercetin)—89.0 µg mL^−1^ (rutin); quercetin was the strongest O_2_^·−^ scavenger (IC_50_ 11 µg mL^−1^), rutin was the least active (81 µg mL^−1^);quercetin was the strongest inhibitor of xanthine oxidase (IC_50_ 15.0 µg mL^−1^), gallic acid – the weakest (112 µg mL^−1^); quercetin had the highest Fe^2+^ chelation capacity (83%), rutin was the weakest chelator (17%); gallic acid showed the highest Fe^3+^ reducing power, expressed as ascorbic acid equivalent, (1081 nM), while rutin was the least potent (46.0 nM). Skalski et al. (2019b) showed in vitro that isorhamnetin, isorhamnetin 3-*O*-glucoside-7-*O*-rhamnoside, and isorhamnetin 3-*O*-β-glucoside-7-*O*-(3-isovaleryl)-α-rhamnoside (**22**) inhibited Fe^2+^/H_2_O_2_-induced lipid peroxidation in human serum, at 5 and 10 µg mL^−1^; the inhibition at 10 µg mL^−1^ was 29.7%, 40.6%, and 29.6%, respectively. In addition, the compounds inhibited the Fe^2+^/H_2_O_2_-induced protein carbonylation (36.1%, 37.6, and 31.0% of inhibition, respectively, 10 µg mL^−1^). Isorhamnetin 3-*O*-glucoside-7-*O*-rhamnoside and **22** also increased the level of the serum thiol groups. Isorhamnetin 3-O-(6-*O*-*E*-sinapoyl-β-D-glucopyranosyl)-(1 → 2)-β-D-glucopyranoside-7-*O*-α-L-rhamnopyranoside (**19**) and isorhamnetin 3-*O*-(6-*O*-*E*-feruloyl-β-D-glucopyranosyl)-(1 → 2)-β-D-glucopyranoside-7-*O*-α-L-rhamnopyranoside (**20**) showed a mild NO^·^ scavenging activity, with EC_50_ values of 208 and 509 µg mL^−1^, respectively (Fang et al. 2013).

### Anti-inflammatory activity

Extracts from fruit of different cultivars of SB efficiently inhibited 15-lipoxygenase (Tkacz et al. 2019). Similarly, extracts from the fruit, leaves and twigs of SB exerted efficient inhibitory activity against synthesis of NO^·^ by LPS-induced RAW264.7 mouse macrophages (Zheng et al. 2019). In a more detailed study, kaempferol 3-*O*-β-D-glucoside-7-*O*-{2-*O*-[2(*E*)-2,6-dimethyl-6-hydroxy-2,7-octadienoyl]}-α-L-rhamnoside (12), kaempferol 3-*O*-β-D-glucoside-7-O-{3-*O*-[2(*E*)-2,6-dimethyl-6-hydroxy-2,7-octadienoyl]}-α-L-rhamnoside (**13**), isorhamnetin 3-*O*-β-D-glucoside-7-*O*-{2-*O*-[2(*E*)-2,6-dimethyl-6-hydroxy-2,7-octadienoyl]}-α-L-rhamnoside (**17**), kaempferol, tiliroside, quercetin, isorhamnetin, ursolic acid (**71**), pomolic acid (**80**), corosolic acid (**77**), and 23-hydroxyursolic acid (**78**) were shown to inhibit synthesis of NO^·^ by LPS and INFγ-induced RAW264.7 cells, with IC_50_ values of 68.8, 67.0, 52.3, 18.2, 26.9, 20.6, 25.1, 17.8, 15.0, 23.6, and 12.5 µM, respectively (Yang et al. 2013). IC_50_ values for the inhibition of NO production were also determined for other compounds isolated from SB fruit, 1,5-dimethyl citrate (97), 2-hydroxy-5-methoxybenzoic acid (**55**), vomifoliol (**94**), dehydrovomifoliol (**95**), 3-*O*-glucosides of syringetin and isorhamnetin: 39.76, 79.39, 74.71, 76.12, 86.33, and 87.01 µM, respectively. The compound **97** was shown to inhibit expression of iNOS and COX-2 in RAW264.7 cells, it suppressed also the production of interleukin 6 and TNF-α (Baek et al. 2020). Oleanolic acid (**70**), maslinic acid (**76**), as well as asiatic acid suppressed the production of NO^·^, iNOS, and interleukin 6 by LPS-induced RAW264.7 cells. Anti-inflammatory effects of these triterpenoid acids were exerted by influencing NF-κB, MAPK, and Nrf2 signalling pathways (Han et al. 2021). The 70% ethanol extract from SB fruit caused a significant reduction of oedema in the rat paw model of acute inflammation. Oleanolic (**70**) and ursolic (**71**) acids were identified as main active constituents of the extract (Rédei et al. 2017).

### Anticoagulant and antiplatelet activity

Butanol extracts from SB leaves and twigs were tested for their influence on human plasma haemostasis, and were found to prolong activated thromboplastin time (APTT) at 50 μg mL^−1^ (Skalski et al. 2019a). Another experiment showed that the phenolic fraction of the SB leaf butanol extract prolonged prothrombin time (PT), and the low-polarity fraction (rich in triterpenoids) of the SB twig butanol extract extended APTT, at the concentration range 0.5–50 µg mL^−1^ (Skalski et al. 2018). In turn, isorhamnetin 3-*O*-β-glucoside-7-*O*-(3-isovaleryl)-α-rhamnoside (22) prolonged thrombin time (TT) in human plasma. Moreover, 22 and isorhamnetin inhibited the thrombin-induced human platelet aggregation at 10 µg mL^−1^, but had no influence on ADP- or collagen-activated platelets (Skalski et al. 2019b). Phenolic and low-polarity fractions of SB leaf and twig butanol extracts inhibited the thrombin-induced adhesion of human platelets to collagen, at 0.5–50 µg mL^−1^. The same preparations inhibited the thrombin- and ADP-induced adhesion of the platelets to fibrinogen, while the phenolic and low polarity fractions from twigs also suppressed the ADP-induced aggregation of platelets. In addition, all tested preparations decreased superoxide anion level, and inhibited lipid peroxidation in thrombin-activated platelets, while they did not inhibit lipid peroxidation in the resting platelets (Skalski et al. 2020). The influence of the total SB fruit flavonoids on vascular endothelial injury was investigated in blood stasis model rats. The flavonoid preparation caused a reduction of whole-blood viscosity, lengthening of PT and APTT at the doses of 0.24 and 0.48 g kg^−1^ b.w., and decreasing of the level of fibrinogen. The reduction of levels of thromboxane B2, 6-keto-prostaglandin F1α, von Willebrand factor (vWF),and thrombomodulin was also observed (Wei et al. 2017).

### Anticancer activity

Anti-proliferative and cytotoxic activity of extracts from fruits of *H. rhamnoides* spp. *yunnanensin*, *sinensis*, *mongolica*, *turkestanica*, and their 15 phenolics were tested on human liver cancer cell line HepG2. The median effective doses (ED_50_) were determined to evaluate their effect for the proliferation of the cells, 0.85, 1.21, 3.03, and 3.31 mg mL^−1^ for extracts from the fruit of spp. *yunnanensin, mongolica, turkestanica*, and *sinensis*, respectively. Isorhamnetin, kaempferol, and quercetin were the most potent proliferation inhibitors (ED_50_ 29.0–80.0 µM), their 3-*O*-sophoroside-7-*O*-rhamnosides of kaempferol and isorhamnetin, as well as isorhamnetin 3-*O*-glucoside-7-*O*-rhamnoside were the least active (ED_50_ > 800 µM). The half maximal cytotoxicity concentrations (CC_50_) of the preparations were usually distinctly higher (e.g. 8.31–16.8 mg mL^−1^ for the extracts, and 76.2- 202 µM for the flavonol aglycones), which indicates that the anticancer activity of the tested preparations may be attributed mainly to their anti-proliferative properties (Guo et al. 2017). In another study, isorhamnetin 3-*O*-glucoside-7-*O*-rhamnoside, isorhamnetin 3-*O*-rutinoside, isorhamnetin 3-*O*-glucoside, and isorhamnetin 3-*O*-glucoside-7-*O*-(3-isovaleryl)-rhamnoside (**22**) showed a weak cytostatic activity against human colon cancer lines; their IC_50_ values against HT-29 cells ranged from 114.2 (isorhamnetin 3-*O*-glucoside-7-*O*-rhamnoside) to > 200 µM (isorhamnetin 3-*O*-glucoside), in a 72 h experiment. In the case of HCT-116 and Caco-2 cells, IC_50_ values for all compounds exceeded 200 µM. In contrast, the phenolic-rich fraction of SB fruit was shown to be moderately active against HT-29 (IC_50_ 47.3 µg mL^−1^), HCT-116 (IC_50_ 81.3 µg mL^−1^), ac Caco-2 (IC_50_ 215.0 µg mL^−1^) cells (Żuchowski et al. 2019). Grey et al. (2010) compared the influence of heptane, ethyl acetate, ethanol, water extracts from SB fruit (prepared successively), as well as ethanol–water fruit extracts (in concentrations up to 2%) on proliferation of HepG2 and Caco-2 cells. The ethanol–water extract, and ethyl acetate extract were the most active proliferation inhibitors. Their activity was dose-dependent, and the ethyl acetate extract had also proapoptotic properties. The activity of the ethyl acetate extract was attributed to its high ursolic acid content. The effect of the ethanol extract from SB leaves on the growth and differentiation of cells of human acute myeloid leukemia (KG-1a, HL60, U937) was also determined. The extract inhibited proliferation of the tested cell lines in a dose-dependent manner, and it showed some cytotoxic activity against HL60, at 100 µg mL^−1^, due to the induction of apoptosis (Zhamanbaeva et al. 2014). Similarly, a hot water extract from SB leaves inhibited proliferation of rat glioma C6 cells in a dose-dependent manner (up to 49.5% inhibition at 62 µg mL^−1^, on the 3^rd^ day). It also reduced viability of the glioma cells (at 0.62–62 µg mL^−1^), while non-cancerous mice NIH/3T3 fibroblast cell line was not affected. The treatment was accompanied by a significant decrease in production of reactive oxygen species in the glioma cells, upregulation of expression of the pro-apoptotic Bax protein, and caused some changes in morphology of nuclei. These reactions suggest that the leaf extract's anti-proliferative properties may be caused by the induction of early events of apoptosis (Kim et al. 2017). In another experiment, SB leaves were sequentially extracted with cyclohexane, hexane, diethyl ether, ethyl acetate, methanol, and water. The water extract turned out to cause a dose-dependent (3.12–50 µg mL^−1^) inhibition of proliferation of C4-2 and LNCaP prostate cancer cells. Moreover, it reduced migration of C4-2, and was shown to inhibit the nuclear translocation of GFP-tagged androgen receptor (AR). Expression of AR and AR-responsive genes was also downregulated (Masoodi et al. 2020). Proanthocyanidins from SB seeds decreased viability of human breast cancer MDA-MB-231 cells (IC_50_ 37.5 μg ml^−1^), inducing apoptosis. The preparation was a potent inhibitor (IC_50_ 0.087 μg mL^−1^) fatty acid synthase (FAS), active in a dose-dependent manner against intracellular FAS in MDA-MB-231 cells (which overexpress the enzyme); the inhibition of intracellular FAS activity induced apoptosis in the cancer cells (Wang et al. 2014). The cytotoxic activity of low polarity fractions of butanol extracts from SB fruit, leaves and twigs were tested against HT29, HCT116, PC3, AGS, MCF7, HS27, and PBMCs cell lines. The determined IC_50_ values were 14.58–74.58 μg ml^−1^ for the twig fraction, 23.33–68.00 μg ml^−1^ for the leaf fraction, and 18.00–42.92 μg ml^−1^ for the fruit fraction, respectively. Further experiments on the most sensitive cell lines (HT29, PC3, AGS) showed that the cytotoxic effect of the tested preparations was caused by induction of apoptosis. Moreover, the fractions had moderate genotoxic activity (the comet assay; up to ~ 16% DNA in tail) at their IC_50_ concentrations. The cytotoxic properties of the low-polarity fractions was attributed to the presence of diverse triterpenoid acids (Marciniak et al. 2021). HRWP-A, a highly methylated homogalacturonan pectin from the fruit of SB significantly inhibited the growth of the Lewis lung carcinoma in mice. It seems HRWP-A did not have any direct cytotoxic properties, but the anticancer effect was due to its immunomodulatory activity. It enhanced the lymphocyte proliferation, augmented the cytotoxicity and phagocytosis of macrophages, increased levels of TNF-α and NO, and stimulated activity of NK cells and cytotoxic lymphocytes in the tumour-bearing mice (Wang et al. 2015).

### Anti-hyperglycemic and anti-hyperlipidemic activity

SB may be useful in treatment of metabolic syndrome and type 2 diabetes and, as shown by in vitro and in vivo experiments. SB methanol leaf extract, its fractions, and six phenolics isolated from the extract (kaempferol 3-*O*-(6-*O*-*E*-*p*-coumaroyl)-glucoside, 1-*O*-*E*-feruloyl-glucose (57), isorhamnetin-3-*O*-glucoside, quercetin 3-*O*-glucoside, quercetin 3-*O*-glucoside-7-*O*-rhamnoside, and isorhamnetin-3-*O*-rutinoside) were tested for their α-glucosidase inhibitory activity, at 0.5, 2.5, and 5 µg mL^−1^. Kaempferol 3-*O*-(6-*O*-*E*-*p*-coumaroyl)-glucoside showed the highest inhibitory activity among all tested pure compounds (at 5 µg mL^−1^), followed by isorhamnetin-3-*O*-rutinoside, isorhamnetin-3-*O*-glucoside, 1-*O*-*E*-feruloyl-glucose, quercetin 3-*O*-glucoside, and quercetin 3-*O*-glucoside-7-*O*-rhamnoside. The highest inhibitory effect was observed (at 5 µg mL^−1^) for the butanol fraction of the crude leaf extract (Kim et al. 2011). α-Glucosidase inhibitors from SB leaves include also the ellagitannin casuarinin (IC_50_ 21 µM) and the saponin arjunglucoside I (IC_50_ 1074 µM; 93) (Yuca et al. 2022). Moreover, five acylated derivatives of kaempferol 3-*O*-soporoside-7-*O*-rhamnoside (1, 2, 4, 9, 10) from seeds of *H. rhamnoides* ssp*. sinensis* were shown to have many-fold higher α-glucosidase inhibitory activity than acarbose, the positive control. Their IC_50_ values ranged between 8 µM (kaempferol-3-*O*-(6-*O*-3,4,5-trimethoxycinnamoyl)-glucoside-(1 → 2)-glucoside-7-*O*-rhamnoside; 2) and 112 µM (kaempferol-3-*O*-[(2*E*)-2,6-dimethyl-6-hydroxy-2,7-octadienoyl(1 → 6)]-glucoside-(1 → 2)-glucoside-7-*O*-rhamnoside; 4) (Li et al. 2019). Inhibition of α-glucosidase (IC_50_ 42–60 mg mL^−1^) and α-amylase (IC_50_ 27–35 mg mL^−1^), as well as lipase (IC_50_ 4–14 mg mL^−1^) was also shown for extracts from the fruit of different cultivars of SB (Tkacz et al. 2019).

Rats with type 2 diabetes, induced by streptozotocin and high-fat diet, were fed with a diet supplemented with a water extract from SB seed residue (400 mg kg^−1^ b.w.), for 6 weeks. The treatment caused a statistically significant reduction of body weight, serum glucose, total cholesterol, and LDL-C levels in the diabetic rats. Their insulin sensitivity index also increased (Zhang et al. 2010). The hypoglycaemic and hypolipidemic effect of the total flavonoids from SB seeds was tested in a high-fat diet fed mouse model. The obese mice were fed with a high-fat chow, supplemented with SB flavonoids (at three doses: 50, 100,150 mg kg^−1^ b.w.), for 12 weeks. A significant reduction of the total body weight, liver weight and epididymal fat pad weight was observed, accompanied by the decrease of the serum total cholesterol, LDL-C, and glucose levels (Wang et al. 2011). Antidiabetic potential of SB juice, and the juice enriched with L-quebrachitol (5 mL daily) was tested on db/db mice in a 10 week experiment. The treatment caused a significant improvement of glucose tolerance and the integrity of pancreatic tissue. The feed intake, body weight gain, blood glucose level, and the expression of insulin receptor β in the liver were also reduced. It seems the presence of L-quebrachitol contributed to the antidiabetic effect of the juice (Xue et al. 2015).

### Antimicrobial activity

Extracts and a phenolic-rich fraction from SB leaves inhibited growth of *Bacillus cereus*, *Pseudomonas aeruginosa*, *Staphylococcus aureus*, *Enterococcus phecalis*, *Escherichia coli*, *Salmonella typhi*, *Shigella dysenteriae*, and *Streptococcus pneumoniae* in the agar-diffusion assay (up to 0.5-1 mg mL^−1^). However, the activities were lower than those for 10 µg mL^−1^ ampicilin (Upadhyay et al. 2010; Yogendra Kumar et al. 2013). Jeong et al. (2010) determined Minimum Inhibitory Concentrations (MIC) against *Bacillus subtilis*, *S. aureus*, *E. coli*, *Salmonella typhimurium*, as well as yeasts *Pichia jadinii* and *Candida albicans*, for methanol extracts from SB roots and stem, and their hexane, ethyl acetate, butanol and water fractions. While MIC values against the bacteria were rarely lower than 500–1000 µg mL^−1^ (except for *S. aureus*), the preparations were more active against the fungi – 125 µg mL^−1^ for preparations from the roots, 62 µg mL^−1^ for the crude extract from the stem, and its hexane and ethyl acetate fractions, and 125 µg mL^−1^ for its butanol and water fractions. Butanol extracts from SB leaves and twigs significantly inhibited growth of *Candida glabrata* G1 (MIC 15.6 and 3.9 µg mL^−1^ for the twig and leaf extract, respectively), and *C. albicans* ATCC 10,231 (MIC 250 and 31.5 µg mL^−1^ for the twig and leaf extract, respectively). Moreover, the extracts had antivirulence properties, inhibiting the formation of biofilm by C. *albicans* ATCC 10,231 (80.6% and 15.3% of inhibition at 0.5 MIC, for the twig and leaf extract, respectively) (Sadowska et al. 2017). In contrast, phenolic fractions and low-polarity fractions from butanol SB leaf extract and butanol twig extract did not inhibit growth of diverse bacteria (*S. aureus*, *Streptococcus mutans*, *B. cereus*, *Lactobacillus acidophilus*, *Helicobacter pylori*, *E. coli*, *Proteus vulgaris*, *P. aeruginosa*), and yeasts (*C. albicans, C. parapsilosis*, *C. krusei*, *C. glabrata*) in a significant degree (MIC mostly 0.5–1.0 mg mL^−1^ or higher). However, the twig and leaf preparations, as well as the phenolic and low polarity fractions of SB fruit extract suppressed the adhesion and formation of biofilm by *S. aureus* and *C. albicans* at 0.5 MIC; the inhibitory activity was generally stronger against *C. albicans*. In the case of *S. aureus*, the investigated low-polarity fractions were more active than the phenolic ones (Różalska et al. 2018).

### Antiviral activity

Ellagitannins from SB leaves were known to be active against influenza and Herpes viruses, while SB leaf extract was found to be active also against dengue virus (Suryakumar and Gupta 2011). In more recent research, 7 phenolic compounds and quinones from SB fruit peels were tested for their antiviral activity. (R)-6,9-dihydroxy-1-oxo-14-noreudesm-5,7,9-triene (63), 2-Hydroxy-1-methoxy-6,9-dioxo-14-noreudesm-1,3,5(10),7-tetraene (64), 1-[3-Methoxy-4-(2-methoxy-4-(1*E*)-propenyl-phenoxy)-phenyl]-propane-1,2-diol (65), and musizin (66) reduced replication of *Herpes simplex* type 2 virus in Vero cells, at 12.5 µM for 63, 65 & 66, and 50.0 µM for 64, as shown by the virus yield reduction assay. The application of a qPCR-based method, to evaluate the level of HSV-2 growth inhibition, enabled to determine IC_50_ values for the tested compounds: 6.25, ~ 25, 12.5, and ~ 12.5 µM for 63, 64, 65, and 66, respectively; musizin-8-*O*-glucoside (67) was also found to be active with IC_50_ ~ 25 µM (Rédei et al. 2019). Isorhamnetin and SB fruit ethanol extract were shown to interact with the human angiotensin-converting enzyme 2 (ACE2), a SARS coronavirus 2 (SARS-CoV-2) receptor. Isorhamnetin also inhibited the entry of SARS-CoV-2 spike pseudotyped virus into ACE2-overexpressing cells (Zhan et al. 2021).

### Other activities

Several flavonolignans from SB fruit exerted immunosuppressive properties. Compounds **44**, **47**, **48**, **33** (cinchonain Ia), and **34** (cinchonain Ib) inhibited the concavalin A-induced proliferation of BALB/c mouse spleen cells, with IC_50_ values of 41.82, 19.42, 20.19, 48.05, and 46.91 µM, respectively. Other compounds, including **43**, **46**, **39** (ent-mururin A), **35** (mururin A), and **36** (mururin B) showed moderate neuroprotective activity. The compounds increased the survival rate of PC12 cells from 50.30% (negative control) to 61.08–71.63%, at 10 µM (Ma et al. 2020). Water–methanol extracts from the fruit of different cultivars of SB exerted moderate anticholinergic activity, their IC_50_ values ranged from 20.16 to 40.60 µg mL^−1^; the samples had also strong inhibitory activity against butylcholinesterase, with IC_50_ < 0.01 µg mL^−1^ (Tkacz et al. 2020). The results suggest that SB fruit may find use in the prevention and treatment some neurological disorders, including neurodegenerative diseases. Ethyl acetate fraction of SB leaf extract increased the viability of neuronal PC-12 cells treated with H_2_O_2_, their membrane integrity (inhibition of lactate dehydrogenase release), and reduced their intracellular ROS level in a dose-dependent manner. The fraction had also some anti-apoptotic properties (Cho et al. 2017). SB fruit juice, administered to Wistar rats with induced epilepsy (at the dose of 1 mL kg^−1^ b.w., for 1 month), significantly reduced the epileptiform activity, improved abnormalities of behaviour, and improved histological structure of cortex and hippocampus (Ladol and Sharma 2021).

Kalemba-Drożdż et al. (2020) investigated the protective effect of different pasteurized fruit juices (bilberry, chokeberry, cranberry, Goji, Noni, rosehips, raspberry, and SB) against DNA damage. Human lymphocytes were incubated with 2-amino-1-methyl- 6-phenylimidazo(4, 5-b)pyridine (PhiP), in presence or absence of 0.1% solutions of the juices. All tested juices efficiently protected lymphocytes against DNA damage induced with 100 µM PhiP. Moreover, the juices inhibited the formation of ROS; it seems the inhibitory activity of the SB juice was higher than those of Noni, cranberry, chokeberry, and raspberry juices. In absence of PhiP, the SB juice did not increase ROS production in lymphocytes, unlike cranberry and Noni juices.

Zhang et al. (2018) tested 46 compounds and groups of compounds isolated from SB fruit, including flavonoids, carotenoids, phospholipids, phenolic acids, aliphatic acids, fatty acids, sterols, for their hepatoprotective activity in rats. Some of them, mainly flavonoids and phenolic acids, inhibited the self-activation of hepatic stellate cells (HSCs), which are known to be engaged in the development of liver fibrosis, with IC_50_ values ranging from 46.03 (isorhamnetin 3-*O*-rutinoside) to 186.34 µM (zeaxanthin dipalmitate). The most active compounds (isorhamnetin 3-*O*-rutinoside, isorhamnetin and protocatechuic acid) were also shown to reduce viability TGF-β-activated HSCs, decreased levels of TNF-α, IL-1, and IL-6 secreted by the them, as well as they induced the HSC cell arrest. Moreover, a vaguely described preparation “active components of SB berries” alleviated rat liver fibrosis (caused by the bile duct ligation) during in vivo experiment. Maheshwari et al. (2011) showed that the ethyl acetate fraction of the 70% ethanol SB leaf extract protected rats from CCl_4_-induced oxidative liver damage. The fraction, administered orally at 25–75 mg kg^−1^ b.w., for 7 days, decreased the serum activity of alanine aminotransferase, aspartate aminotransferase, and bilirubin level. The treatment prevented the depletion of hepatic antioxidant enzymes (SOD, catalase, glutathione peroxidase, glutathione reductase), and glutathione level. It also inhibited lipid peroxidation and protein carbonylation, as well as prevented histopathological changes in rat livers.

4-[(*E*)-*p*-coumaroylamino]butan-1-ol (**99**), and a pyridoindole alkaloid hippophamide (**101**) showed cardioprotective properties, decreasing the doxorubicin-induced cytotoxicity in H9c2 embryonic rat cardiac cells, at concentrations 5–160 µM; 101 was a more active compound. They significantly reduced the intracellular ROS levels (10–40 µM) in a dose-dependent manner. The compounds inhibited the doxorubicin-induced apoptosis, i.a. by suppressing cleaved-caspase-3 protein expression, suppressing the activation of c-Jun N-terminal kinases, increasing ATP level (mainly 101), and decreasing damage to mitochondrial DNA (only 101) (Zhou et al. 2021).

### Clinical trials

In the years 2010–2021, different preparations from SB were subjected to at least several clinical trial studies. Lehtonen et al. (2010) investigated the influence of a crushed SB fruit, an oil-free SB fruit (after the supercritical CO_2_ extraction), and ethanol-extracted oil-free fruit on postprandial hyperglycemia and insulin response. Each of the ten healthy male volunteers consumed breakfasts of yogurt + 50 g glucose, containing the above mentioned supplements and a placebo on consecutive days of the experiment, in a randomized order. The amounts of the fruit preparations corresponded to 200 g of fresh SB fruit. The trial showed that the SB fruit and the oil-free fruit significantly suppressed the postprandial peak insulin response, and stabilized postprandial hyperglycemia and subsequent hypoglycemia. The third preparation was not active. The results suggest that the SB oil, containing carotenoids, phytosterols, and tocopherols had a minor influence on the investigated parameters, and ethanol-soluble compounds, possibly flavonoids and other phenolics, were responsible for the observed antidiabetic effect. In the article of Lehtonen et al. (2011), the dried SB fruit, as well as SB oil (supercritical CO_2_-extracted), an ethanol extract from the oil-free SB fruit (spray-dried in a 1:1 mixture with maltodextrin), and frozen bilberries were tested for their ability to alleviate problems associated with obesity and metabolic syndrome, in an experiment with randomized cross-over study design. These fruit preparations were consumed by 80 female volunteers for 33–35 days (intervention periods), in an independently randomized order; the intervention periods were separated by 30–39-day wash-out periods. Doses of the fruit preparations were supposed to be equivalents of 100 g of fresh berries. The consumption of SB fruit, and SB oil caused a small, but statistically significant reduction of waist circumference. The SB fruit reduced also the serum glucose and TNFα levels; the latter parameter was also decreased by the ethanol extract. Another publication, based on the same or a very similar experiment, provided some metabolomic data about the participants’ serum samples. The SB fruit, and (particularly) SB oil tended to improve serum lipid parameters (e.g. lowering serum cholesterol, LDL-C, serum triglycerides), though the observed changes were usually not statistically significant. The positive effects were the best seen in participants who had a higher cardiometabolic risk at the beginning of the trial (Larmo et al. 2013). Vashishtha et al. (2017) investigated the influence of the SB seed oil (supercritical CO_2_-extracted; 0.75 mL per day, for 30 days) on cardiovascular risk factors in people with hypertension in the randomized, controlled, double blind longitudinal study (106 volunteers, three groups: I – the healthy taking the oil, II – hypertensive persons taking the oil, and III – hypertensive persons taking a placebo, sunflower oil). The treatment caused a statistically significant reduction of serum LDL-C, total cholesterol, oxidized LDL, and triglycerides, more visible in people with hypertension. In contrast, the consumption of SB fruit puree (a double-blind, randomized, placebo-controlled trial; 111 volunteers with hypercholesterolemia, two groups; 90 mL of the puree or placebo per day, for 90 days) did not improve the serum lipid parameters. However, diastolic blood pressure was significantly decreased. In addition, the SB treatment tended to have an anti-inflammatory effect (a reduction of levels of hypersensitive C-reactive protein and soluble vascular cell adhesion molecule-1) (Zhou et al. 2020). Rodhe et al. (2013) evaluated the effect of the supercritical CO_2_-extracted SB oil on oral health, inflammation, and DNA damage in haemodialysis patients by a randomized, double-blinded, and placebo-controlled crossover study. The experiment comprised two treatment periods (for the oil and placebo, dosed 2 g per day; 8 weeks) separated by a wash-out period. The oil did not have any significant influence on the investigated parameters. The effect of the consumption of a SB oil on vaginal atrophy in postmenopausal women was tested in a placebo-controlled, randomized, double-blind, study (96 volunteers; two groups; 3 g of SB oil or placebo per day, for 3 months). A significantly better rate of the improvement in the integrity of vaginal epithelium was observed in the SB oil group, as compared to the placebo group. The treatment did not significantly influenced serum lipid parameters (Larmo et al. 2014). A SB emulsion (with its juice and oil) was shown to alleviate symptoms of functional dyspepsia in children. The study comprised 120 children (11 months – 10 years), divided into three groups: I – a group treated with the SB emulsion; II – a group treated with domperidone (a dopamine antagonist, used in treatment of nausea); III – a group treated with the SSB emulsion and domperidone. The SB emulsion was a daily doses of 5–15 g, depending on the children’s age, for 8 weeks. The application of the SB emulsion significantly improved appetite factors, increased levels of leptine and neuropeptide Y, and improved gastric emptying, gastrointestinal digestive functions, as well as children’s growth and development (Xiao et al. 2013). Gao et al. (2014) investigated effectiveness of sea buckthorn capsules (Hebei Shengxing Seabuckthorn Pharmaceuticals, Shijiazhuang City, China) in treatment of nonalcoholic fatty liver disease. Patients (94) were divided into two groups: a treatment group, taking the SB capsules (daily dose—4.5 g, for 90 days), and a control group, taking placebo capsules. At the end of the experiment, the SB treatment caused a statistically significant reduction of levels of serum alanine aminotransferase, LDL-C, triglycerides, collagen type IV, and hyaluronic acid, as compared to the control group. Liver stiffness measurement, and liver/spleen ratio were also improved. Sea buckthorn (especially the oils) is well known for its wound and burn-healing properties. Abdullahzadeh and Shafiee (2021) compared the effectiveness of a cream containing 40% of “the active ingredient of sea buckthorn” fruit in treatment of second-degree burns, with that of a 1% silver sulfadiazine (SSD, a topical medicine commonly used for treatment of burns) cream. A randomized triple-blind clinical trial comprised 55 hospital patients. The sea buckthorn-based preparation significantly shortened the period of burn healing, as compared to the 1% SSD cream.

### Toxicity studies

Acute and sub-chronic toxicity of SB fruit oil was evaluated on mice and rats, respectively. The oil, obtained from the ground dried fruit by supercritical CO_2_ extraction, was shown to contain isorhamnetin (0.12 mg g^−1^). In the acute toxicity test, the oil was administered by gavage, in a single dose (at 20 mL kg^−1^ b.w.). After 14 days, there was no mortality among the mice, and no sign of toxicity was observed in their organs. In the sub-chronic toxicity test, the oil was given to rats at 2.5, 5, and 10 mL kg^−1^ b.w., for 90 days. No significant changes in haematology and serum biochemistry were observed. Administration of the oil did not significantly influence food consumption and body weight gains, the weight of organs or their histology. The maximum tolerated dose of SB oil was determined as > 20 mL kg^−1^ b.w. for mice, and the no-observed-adverse-effect-level (NOAEL) was > 10 mL kg^−1^ b.w. for rats (Zhao et al. 2017). Genotoxicity and teratogenicity of the same (or similar) oil was also determined, using mice and rats. Genotoxicity tests comprised the mice sperm abnormality test (the oil doses 2.34, 4.68, and 9.36 g kg^−1^ b.w., for 5 days), the in vivo mice bone marrow micronucleus test (the same oil doses, for 2 days), as well as the bacterial reverse mutation assay (*Salmonella typhimurium*; 8–5000 µg of the oil per plate). Teratogenicity of the oil was determined on rats (the oil doses 1.17, 2.34, 4.68 g kg^−1^ b.w., for 10 days). The tested SB oil was not genotoxic or teratogenic under the applied experimental conditions (Wen et al. 2020). In another experiment, SB seed oil was administered intramuscularly to rabbits (0.5, 1, 1.5 mL kg^−1^ b.w., once a week, for 7 weeks). A dose-dependent increase in the total serum antioxidant capacity was observed with maximum 48 h after the oil injection. The treatment did not significantly influence haematological parameters and serum biochemistry, no significant histopathological changes were observed in organs (Ali et al. 2011). Tulsawani (2010) investigated the influence of the water extract from SB fruit on rats, in a 90 day experiment. The extract was administered by gavage, at the dose of 100, 250 and 500 mg kg^−1^ b.w. No toxic effect was observed. However, an increase in serum glucose at 250 and 500 mg kg^−1^ doses was detected at the end of the experiment, and 100 mg kg^−1^ b.w per day was determined as the NOAEL for the extract. A herbal antioxidant supplement, containing SB pulp and “extract of indigenous medicinal plants of high altitudes that tone up the gastric and intestinal functions” was orally administered to mice at 2, 4, 8, and 10 g kg^−1^ b.w., to test its acute toxicity. During 14 days, no change in mice behaviour and body weight gains were observed. To determine sub-acute toxicity of the preparation, 2, 4, and 8 g kg^−1^ b.w. doses were administrated daily, for one month. No change in the animal behaviour, body weight gains, haematological and biochemical parameters of blood, as well as organ histology was observed (Ali et al. 2012). The above results seem to confirm that SB oils and fruit extracts are generally safe food additives.

## Conclusion

Sea buckthorn is currently a well-known plant. Its vitamin-rich fruit is more and more frequently consumed, usually in processed form, seed and fruit pulp oils are used in the cosmetic industry, and medicine. The plant is listed in the Chinese Pharmacopoeia, and its different parts are used in the traditional medicine of China, Tibet, Mongolia, and other countries of central and eastern Asia. Health-promoting and medicinal properties of SB caused a large scientific interest in this plant. As it has been shown in this review, The years 2010–2021 brought significant progress in phytochemical research on SB. At least 24 new flavonoids, 10 flavonolignans, 6 saponins, 3 alkaloids, two sesquiterpenoids, one ellagitannin, and one phenylpropanoid derivative were isolated during this period from SB fruit, seeds, and leaves. In addition, dozens of known specialized metabolites were also isolated, some of them for the first time from this plant. Summarizing, SB is a rich source of natural products, including flavonoids, phenolic acids, ellagitannins, triterpenoids, phytosterols, carotenoids, and volatile compounds. Flavonolignans, lignans, sesquiterpenoids, naphthalenes, anthraquinonoids, triterpenoid saponins, alkaloids or carboxylic acid derivatives were also found. These compounds undoubtedly significantly contribute to medicinal properties of SB. During the period in question, diverse pharmacological effects of SB extracts and natural products were extensively investigated, including antioxidant, anti-inflammatory, anticancer, antidiabetic, antithrombotic, antimicrobial, antiviral, immunosuppressive, neuroprotective activity, and other effects. Casuarinin, one of the main ellagitannins of SB leaves, seems to be most promising compound, a potent α-glucosidase inhibitor and free radical scavenger, potentially useful in treatment of diabetes. Sea buckthorn flavonolignans, reported for the first time from the plant, also constitute an interesting group of phytochemicals. Among them, compounds **47** and **48** had significant immunosuppressive properties, while **43** and **46** showed distinct neuroprotective activity in in vitro tests.

The reviewed publications suggest that preparations made from different parts of SB may be potentially useful in the prevention and treatment of neoplasms, metabolic syndrome, diabetes, and other inflammation- and oxidative stress-related disorders and diseases, or as anti-virulence agents, decreasing the formation of biofilm by pathogenic microbiota. In the author’s opinion, future research should pay more attention to SB triterpenoids, triterpenoid saponins, and ellagitannins. Though ellagitannins seem to be the dominant phenolics of SB leaves, considerable amounts of saponins can be found in the seeds and leaves, and triterpenoids occur in all parts of this plant, these compounds were relatively rarely investigated (especially ellagitannins and saponins), as compared to SB flavonoids, phenolic acids, or carotenoids. Triterpenoids, saponins, and ellagitannins are known for their bioactivity, and undoubtedly significantly contribute to the pharmaceutical properties of sea buckthorn.

## Supplementary information

A list of sea buckthorn specialized metabolites (with their molecular formulas, exact masses, PubChem CID numbers, CAS numbers, and SMILES strings) is provided as a supplementary file.

## Abbreviations

ABTS: 2,2′-Azino-bis(3-ethylbenzothiazoline-6-sulfonic acid)
b.w: Body weight
COX: Cyclooxygenase
DPPH: 2,2-Diphenyl-1-picrylhydrazyl
EC_50_: The half maximal effective concentration
ED_50_: Median effective dose
IC_50_: The half maximal inhibitory concentration
IL: Interleukin
iNOS: Inducible nitric oxide synthase
LC-HRMS: Liquid chromatography-high resolution mass spectrometry
LDL-C: Low-density lipoprotein cholesterol
LPS: Lipopolysaccharide
MAPK: Mitogen-activated protein kinase
MIC: Minimum inhibitory concentration
NF-κB: Nuclear factor kappa B
NO: Nitric oxide
NOAEL: No-observed-adverse-effect-level
Nrf2: Nuclear factor erythroid 2-related factor 2
ROS: Reactive oxygen species
SB: Sea buckthorn
TEAC: Trolox equivalent antioxidant capacity
TGF-β: Transforming growth factor beta
TNF-α: Tumour necrosis factor alpha
